# TEA-LWNet: a lightweight multispectral feature enhancement network based on RGB–NIR fusion for detection of tea leaf diseases and pests

**DOI:** 10.3389/fpls.2026.1879101

**Published:** 2026-08-28

**Authors:** Zhenyun Wang, Fang Wang, Haifeng Lin

**Affiliations:** 1 College of Information Science and Technology, Nanjing Forestry University, Nanjing, China; 2 College of Electronic Engineering, Nanjing Xiaozhuang University, Nanjing, China

**Keywords:** deep learning, lightweight detection, RGB–NIR image, small target detection, tea pest and disease detection

## Abstract

To address the low contrast and mild blur in aerial tea plantation imagery, this study introduces an RGB–NIR dataset composed of UAV-acquired RGB and near-infrared images. The RGB–NIR representation provides complementary visible and near-infrared information, enhancing the contrast between damaged and healthy leaf regions while reducing interference caused by shadows and highlights. This improves the separability of small pest-damaged areas and early disease lesions in weakly textured tea canopy scenes. Based on this, we propose TEA-LWNet, a lightweight multi-scale detection framework. Its backbone, Multi-FENet, leverages a DS-MBS module for simultaneous high-order semantic expansion and low-level detail preservation. The neck features an Adaptive Enhancement (AE) module for spatial-semantic alignment and an MDFF structure with embedded Gated Fusion (GF) to suppress background diffusion during upsampling. Optimization is driven by SAB-Loss—combining IoU, Distributed Focal Loss, and BCE—enforcing scale-invariant geometric constraints and sub-pixel boundary calibration to prioritize small target learning. Evaluations on a self-built RGB–NIR dataset demonstrate that TEA-LWNet achieves an mAP@0.5 of 93.48% under strict parameter and FLOP constraints. Ablation studies confirm the functional complementarity of the proposed modules in cross-layer noise suppression and boundary refinement. Overall, TEA-LWNet achieves a favorable balance between detection accuracy and model complexity, indicating its potential for resource-constrained agricultural monitoring systems. However, its runtime performance on UAV or embedded edge hardware remains to be further validated.

## 1 Introduction

Tea (Camellia sinensis), an important economic crop with a long history of cultivation, occupies a significant position in global agricultural trade and cultural exchange. Modern research indicates that tea contains various bioactive components such as polyphenols, amino acids, and alkaloids, which have been confirmed to possess physiological functions like antioxidants, anti-inflammatory, and neuroprotective effects. These characteristics contribute to the global consumption of tea and support its economic importance in agriculture. However, phytopathological surveys in recent years show that pest and disease problems in tea trees are increasingly prominent. In particular, the combined infestation of Apolygus lucorum (Xiong et al., 2024) and Tea Leaf Blight (TLB) poses a serious threat to tea yield and quality (Hu et al., 2024).

Apolygus lucorum, belonging to the Hemiptera order and Miridae family, is a major pest in tea plantations and orchards in the Yangtze and Yellow River basins of China. Its host range is extensive, including economic crops such as tea, pear, peach, and jujube trees. Apolygus lucorum uses its piercing-sucking mouthparts to feed on the tender leaves and buds of tea trees, leading to symptoms like irregular perforations and damaged leaf margins. Photosynthetic efficiency in affected tea trees decreases, new shoot growth is inhibited, ultimately resulting in a significant drop in tea yield. The finished tea also suffers severely in quality, with a bland taste and increased astringency. This pest reproduces extremely rapidly in high-temperature and high-humidity environments. If not controlled in time, it can escalate into a severe outbreak in a short period, causing major economic losses to tea plantations. Since the 1980s, the expansion of agricultural irrigation in China has led to a general increase in field humidity, providing a suitable habitat for the reproduction of Apolygus lucorum, causing its range and severity of damage to intensify annually. Meanwhile, TLB, a foliar disease caused by pathogenic fungi, often peaks during the rainy season and spreads rapidly. This disease not only reduces the photosynthetic capacity of tea trees but, in severe cases, leads to premature leaf senescence and abscission, disrupting the ecological balance of the tea plantation and consequently affecting the sustainable development of the tea industry. Given the dual threat that the combined infestation of Apolygus lucorum and TLB poses to both the ecological and economic structures of China’s tea plantations, the formulation and implementation of early comprehensive control measures are imperative.

Before the popularization of deep learning technology, the identification of tea pests and diseases primarily relied on manual experience and handcrafted feature engineering. This paradigm typically involved generating candidate regions through methods like threshold segmentation or vegetation indices, followed by extracting explicit descriptors such as color moments, Gray-Level Co-occurrence Matrix, Local Binary Patterns, or shape moments. Finally, classifiers like Support Vector Machines (Mohammadi et al., 2024) or Random Forests were used for discrimination. This traditional paradigm had the advantages of simple implementation, strong interpretability, and low computational resource requirements. However, its limitations were also significant: such methods are highly sensitive to imaging conditions, making it difficult to effectively characterize small targets and early weak symptoms; their robustness across different scales and viewpoints is insufficient; simultaneously, feature engineering relies heavily on heuristic rules, leading to limited generalization ability of the models.

After the popularization of deep learning, introducing Convolutional Neural Networks (CNNs) (Bhuyan et al., 2024) into the tea pest and disease monitoring and warning system for optimization, and promoting them synergistically with green control strategies like biological control and ecological regulation, can both reduce reliance on chemical pesticides and enhance ecological stability. It also provides a feasible pathway to ensure yield and quality while reducing economic losses. In recent years, significant progress in image processing and deep learning technologies has shown immense potential in their widespread application for object recognition.

In the context of tea pests and diseases, existing research has continuously pushed network structures from basic CNNs toward more expressive architectures. For example, Bao et al. used AX-RetinaNet (Bao et al., 2022), which enhances the backbone-to-neck information flow with an X-module, for the automated detection of tea shoot blight and white powdery mildew in natural scenes. It can suppress redundant feature interference to some extent, but accuracy improvement remains limited. Then introduced mechanisms like dual-dimensional hybrid attention into an integrated detection framework to form the DDMA model (Bao et al., 2023), reporting better recognition performance for TLB, but the overall structure still has room for further compression. While detection frameworks continue to iterate, lightweight design has become a core demand: Xia et al. used MobileNeXt modules for a compact design of the detection backbone, enhancing representation capability for multiple common tea diseases (Xia et al., 2024), but challenges remain for small-scale lesions. Ye et al. specifically modified networks for small target detection, improving the detection capability for small targets (Ye et al., 2024a), but deficiencies in training efficiency and model lightweighting still affect its practical deployment potential (Yin et al., 2024)On a more macro level of representation paradigms, self-attention has provided new paths for modeling long-range dependencies; hybrid designs integrate convolutions into multi-level Transformers or introduce lightweight attention into convolutional networks to strike a balance between local priors and global modeling. The above works outline a clear problem landscape: simply stacking heavier attention modules or increasing width and depth can push the performance ceiling but brings significant increases in parameters and FLOPs; conversely, unilaterally compressing the model easily sacrifices multi-scale expression and small-target separability.

All the above methods only utilize RGB images for learning and detection. However, the representation of RGB images is limited to the three visible light channels, resulting in limited spectral discriminability. This representation shows obvious inadequacy in sensing subtle changes in plant physiological states, such as fluctuations in chlorophyll content or tissue water content. In the high-texture background of tea plantation foliage, non-uniform irradiation caused by shadows, specular highlights, and background soil significantly alters the apparent color distribution of targets. Meanwhile, the high inter-channel correlation among the three RGB channels introduces significant information redundancy, limiting its potential in enhancing feature separability. Therefore, under complex conditions such as low contrast, backlighting, or partial occlusion, detection paradigms relying solely on RGB color features are highly prone to high rates of false positives and false negatives. This limitation makes it difficult to stably support the practical needs of fine-grained small target detection and early warning.

Compared with RGB-only images, NIR information provides complementary cues beyond visible color and texture. RGB images mainly describe the surface appearance of tea leaves, whereas NIR responses are more closely related to internal tissue properties, water status, and material composition. Recent multispectral studies have provided experimental evidence for the sensitivity of NIR responses to plant stress. Duan et al. reported that wavelengths in the 780–1000 nm range, including bands near 850 nm, contributed to the early detection of pepper Phytophthora blight, while Lauretti et al. demonstrated that VIS–NIR measurements in the 705–962 nm range could discriminate different levels of salinity stress in tobacco plants (Duan et al., 2024). Previous tea-related studies have shown that NIR spectroscopy can effectively capture composition- and quality-related differences in tea samples. For example, Yang et al. transformed NIR spectral data into pseudo images and used CNN-based models for tea quality classification across multiple tea brands and grades, demonstrating that NIR signals contain discriminative information related to tea sample composition and quality. At the canopy scale, recent UAV studies have also demonstrated the value of combining RGB imagery with NIR-containing multispectral information for disease and pest recognition. Li et al. fused RGB images with vegetation-index representations derived from multispectral bands including 840 nm for apple disease and pest classification, whereas Mora et al. used co-registered RGB and MicaSense RedEdge imagery with HIS pansharpening for banana Xanthomonas wilt detection (Mora et al., 2024). More specifically, quantitative spectroscopy studies on tea leaves indicate that foliar diseases and piercing-sucking insect damage can alter reflectance in the red-edge and near-infrared regions. Mao et al. (2024) identified 693, 727, and 766 nm as sensitive bands for the early detection of tea gray blight, achieving an accuracy of 92.6% under controlled conditions and 87.8% under field conditions using the 693 and 727 nm bands. Wu et al. (2025) further reported severity-dependent spectral differences in tea red scab over the 718–932 nm range. For piercing-sucking insect damage, Xu et al. (2025) observed distinguishable reflectance curves among different tea green leafhopper damage levels and selected informative bands within 780–866 nm, which partially overlap the 834–886 nm passband of the NIR sensor used in this study. For *Apolygus lucorum* specifically, Xie et al. (2026) found that damage severity was significantly associated with leaf color, texture, and surface traits; amino acid and epicatechin contents decreased, whereas gallocatechin gallate increased with increasing damage severity. These findings provide tea-specific physiological and spectral support for introducing the 860 ± 26 nm image as a complementary channel. Nevertheless, these studies are not exact substitutes for spectral measurements of the TLB and *A. lucorum* samples used in the present study. Therefore, the 860 nm band is not interpreted as a target-specific diagnostic wavelength.

Therefore, this study introduces an RGB–NIR fusion representation to compensate for the accuracy reduction caused by model lightweighting. RGB and NIR images were acquired in tea plantation environments and formed into a four-channel input after geometric registration and radiometric normalization. Based on this input representation, a lightweight detection framework, TEA-LWNet, is proposed for Apolygus lucorum and TLB detection in tea plantation environments. The data were collected by the authors in real plantation environments across multiple time periods, lighting conditions, and backgrounds. After RGB–NIR preprocessing and bounding box annotation, the dataset was used for model training and evaluation. The network design follows the co-optimization principle of accuracy and efficiency, strengthening feature representation under weak contrast and complex backgrounds while maintaining a lightweight structure. Driven by the combination of RGB–NIR fusion and a compact detection framework, TEA-LWNet improves the recognition of Apolygus lucorum and TLB without substantially increasing computational overhead, providing a practical technical pathway for early warning in tea plantations.

TEA-LWNet is designed around “channel, scale redistribution, and path-level aggregation”: The Multi-Feature Extraction Network (Multi-FENet) serves as the lightweight backbone, using convolution and depthwise separable convolution paired with linear projection to achieve progressive downsampling (Hogräfer et al., 2022) and channel compression, obtaining hierarchical features B3, B4, B5. AE acts as the core mechanism of the neck, performing spatial alignment and semantic calibration on the multi-level features. Through high- and low-order channel decomposition and learnable weight redistribution, it suppresses channel redundancy and highlights discriminative dimensions, enhancing multi-scale semantic consistency and fusibility. Multimodal Deep Feature Fusion (MDFF) constructs bidirectional (top-down and bottom-up) feature paths, completing cross-layer interaction through lateral connections, concatenation, and lightweight convolution. This forms a fine-grained B5 (beneficial for larger targets), a balanced B4 (semantic and structural), and a strong-semantic B3 (beneficial for small targets). A three-branch detection head then outputs categories and bounding boxes in parallel. Compared to existing practices, the innovations of TEA-LWNet are: using channel-scale adaptive redistribution to directionally alleviate the fusion conflict between shallow-layer texture and deep-layer semantics within a limited computation budget; reconstructing multi-scale consistency through bidirectional path aggregation without significantly increasing parameters and FLOPs; and focusing structural overhead on the most beneficial links through lightweight convolution and linear projection, thereby achieving a better balance between accuracy improvement and controllable model size.

In summary, TEA-LWNet extends the effective experience of end-to-end detection in image quality enhancement, pyramid fusion, and attention mechanisms. It also borrows ideas from self-attention and hybrid representations but highlights its advantage—“lightweighting” through a more computation-budget-friendly structural path. We will validate the comprehensive advantages of TEA-LWNet and the Apolygus lucorum—TLB thematic RGB-NIR multispectral dataset from the three dimensions of overall accuracy, small target detection, and model complexity.

## 2 Materials

### 2.1 Data collection

From March to June 2025, RGB and near-infrared (NIR) images of tea diseases and pests were collected from tea plantations in Ya’an, Sichuan Province, China. Image acquisition was performed using a DJI Mavic 3M (Mavic 3 Multispectral, DJI, Shenzhen, China), which integrates one RGB camera and four multispectral cameras. In this study, the RGB images and the NIR-band images centered at 860 ± 26 nm were used to construct the multispectral dataset. The RGB camera is equipped with a 20-MP 4/3-inch CMOS sensor, whereas the NIR camera uses a 5-MP 1/2.8-inch CMOS sensor. The main specifications and functional roles of the two imaging sensors are summarized in **Table 1**.

**Table 1:** Specifications and functional roles of the RGB and NIR imaging sensors integrated into the DJI Mavic 3M.

Sensor/model	Spectral range or band	Imaging specifications	Primary role
NIR band of the DJI Mavic 3M multispectral camera	Central wavelength: 860 ± 26 nm	1/2.8-inch CMOS; 5 MP; FOV: 73.91°; equivalent focal length: 25 mm; aperture: f/2.0; fixed focus; gain range: 1×–32×; electronic shutter: 1/30–1/12800 s; maximum image size: 2592 × 1944 pixels; TIFF format	Measures tea-canopy reflectance within 834–886 nm and provides a physically acquired complementary input to RGB; target-specific diagnostic sensitivity at 860 nm was not directly measured in this study
RGB camera of the DJI Mavic 3M	Visible spectrum, approximately 400–700 nm	4/3 CMOS; 20 MP; FOV: 84°; equivalent focal length: 24 mm; aperture: f/2.8–f/11; ISO: 100–6400; electronic shutter: 8–1/8000 s; mechanical shutter: 8–1/2000 s; maximum image size: 5280 × 3956 pixels; JPEG/DNG format	Provides visible-spectrum color, texture, edge, and high-resolution spatial information for target localization and recognition.

Low-altitude UAVs operating in tea plantations are susceptible to pitch vibrations, leaf canopy occlusion, and alternating highlight/shadow conditions. RGB imagery often exhibits insufficient local contrast and relaxed structural boundaries. The visibility of small lesions and tiny insect holes decreases significantly under motion blur (Ismagilov et al., 2025) and limited depth-of-field conditions. In the present dataset, the registered 860 ± 26 nm image supplied a physically measured complementary channel, and the fused images showed improved visual contrast for some damaged regions under complex illumination. This is treated as an empirical image-level observation. Because leaf-level reflectance curves and biochemical variables were not measured, the observed contrast cannot be uniquely attributed to changes in water status, pigment content, or tissue structure. In this study, paired RGB and NIR images were used to construct the RGB–NIR input representation. The preprocessing steps are as follows:

First, the RGB and NIR images were resized to a unified spatial resolution. Because the RGB and NIR channels may have slight spatial offsets caused by different imaging centers or optical paths, geometric registration was performed before fusion. In this study, the NIR image was aligned to the RGB image before fusion. Corresponding control points were selected from stable canopy structures, leaf margins, and visible lesion or damage boundaries. A geometric transformation was then estimated to warp the NIR image into the RGB coordinate system. After registration, radiometric normalization was applied to reduce illumination variation across different acquisition periods. The NIR image was aligned to the RGB image to ensure spatial correspondence between visible texture and near-infrared response. Radiometric normalization was then applied to reduce illumination variation across different acquisition periods. After registration and radiometric normalization, the RGB image and the registered NIR image were fused using the HSV-Brovey strategy. The resulting HSV representation was subsequently converted back into a three-channel RGB-like image, which served as the actual input to TEA-LWNet, as defined in Equation 1:

(1) $${\text{I}}_{input}={\text{I}}_{HSV-Brovey}\in {\mathbb{R}}^{H\times W\times 3}$$

Where ${\text{I}}_{HSV-Brovey}$ denotes the final three-channel RGB-like image generated from the registered RGB and NIR images. TEA-LWNet does not directly receive a four-channel RGB-NIR tensor.

In the fusion process, the registered NIR image was used as a complementary spectral channel to the RGB image. Compared with RGB channels, the NIR channel can provide additional responses related to leaf tissue structure, water status, and background reflectance differences. This helps suppress part of the interference caused by shadows, highlights, and complex leaf textures, thereby improving the visibility of small damaged regions and early disease lesions. The NIR channel used in this study was acquired from near-infrared imaging data and was treated as a physically measured spectral band rather than a channel generated from RGB images.

The NIR channel was fused with the visible-light image. First, both the RGB and NIR images were resized and cropped to 640×640pixels. Then, after geometric registration and normalization, the RGB image and the registered NIR image were fused in HSV space using the Brovey-based strategy described below (Mora et al., 2024). Related UAV disease studies have also employed intensity-based pansharpening to integrate co-registered RGB and multispectral imagery. The fusion operation is defined in Equation 2:

(2) $$V^{\text{\textbackslash{}*}}=(1-\alpha )V+\alpha \;\hat{NIR}$$

V is the Value (brightness) channel in the HSV color space, representing the pixel’s illumination intensity, without changing the Hue and Saturation. Mathematically, if R,G,B∈[0,1], the value channel is calculated according to Equation 3:

(3) $$\begin{array}{c} V=\max(R,G,B) \end{array}$$

In OpenCV’s 8-bit HSV representation, V∈[0,255]; in float representation, V∈[0,1]. The brightness cue is injected, and the Brovey transform is used as a control (Medina et al., 2019).

It should be emphasized that HSV-Brovey fusion is a deterministic preprocessing strategy rather than a learnable component of TEA-LWNet. The registered NIR response is incorporated into the value channel before model training and inference, and the resulting HSV representation is converted back into a three-channel RGB-like image. Therefore, this fusion process introduces no additional trainable parameters into the detection network and is used not only for visual enhancement but also to generate the actual input images for model training and inference.

**Figure 1:**
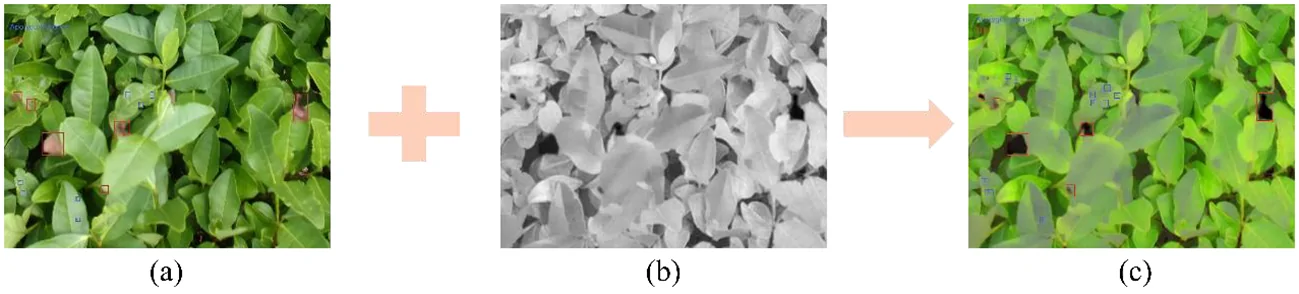
Visual comparison of input representations. **(a)** RGB image. **(b)** NIR image. **(c)** RGB–NIR fused image.

**Figure 1** indicates that the RGB–NIR fused image exhibits clearer contrast between damaged regions and healthy leaf backgrounds than the original RGB image. The fused representation provides additional measured contrast, allowing leaf contours, lesion regions, and small pest-damaged areas to remain more distinguishable under occlusion and uneven illumination. Because leaf-level spectra and biochemical variables were not measured, this visual difference is not interpreted as direct evidence of changes in tissue structure or water status. For Apolygus lucorum damage, the fused representation enhances small punctate feeding traces, local perforations, and damaged leaf margins. For TLB, it improves the visibility of larger irregular lesions, gradual color transitions, and blurred lesion boundaries. Therefore, the RGB–NIR representation helps reduce missed detections and false positives caused by shadows, leaf veins, and complex canopy textures.

For the subsequent data construction phase, this clearer target presentation helps define annotation boundaries and class attributes, reduces annotation ambiguity, and provides more reliable label quality and more stable visibility of small targets for the training set.

Finally, the LabelImg tool was used to manually annotate the dataset (Ke et al., 2024). The annotation results were saved in XML file format, which can clearly represent the hierarchical structure and attribute information of the data, facilitating subsequent parsing and analysis. The dataset in XML format was then converted to TXT. Finally, the dataset was split into training, test, and validation at a ratio of 8:1:1.

#### 2.1.1 RGB-NIR registration accuracy

To quantitatively evaluate the reliability of RGB-NIR registration, 50 paired RGB and NIR images were randomly selected from different acquisition sites, viewing angles, and illumination conditions. For each image pair, corresponding control points were identified on stable visual structures, including leaf margins, canopy edges, and lesion or pest-damage boundaries. The registration error was calculated as the Euclidean distance between the corresponding control points after alignment.

As shown in **Table 2**, the average registration error was 0.84 pixels, with an RMSE of 1.07 pixels and a median error of 0.72 pixels. In addition, 73.5% of the control points had an error below 1 pixel, and 94.1% had an error below 2 pixels. The maximum error was 3.26 pixels, which mainly occurred in image pairs with strong canopy occlusion or slight leaf motion. These results indicate that the RGB and NIR images were generally well aligned before fusion. Nevertheless, sub-pixel and pixel-level residual misalignment may still exist in local regions, which should be considered when designing the fusion strategy for small-target detection.

**Table 2:** Quantitative evaluation of RGB-NIR registration accuracy.

Metric	Value
Number of image pairs	50
Number of control points	612
Mean registration error/pixel	0.84
RMSE/pixel	1.07
Median error/pixel	0.72
Standard deviation/pixel	0.66
Maximum error/pixel	3.26
Control points with error < 1 pixel/%	73.5
Control points with error < 2 pixels/%	94.1

Nevertheless, slight local misalignment may still occur in UAV-acquired tea canopy images due to leaf motion, canopy occlusion, and minor viewpoint differences. Such residual misalignment may affect very small targets, including tiny feeding traces and early lesion boundaries. Therefore, the influence of residual misalignment was considered when selecting the fusion strategy, as discussed below.

### 2.2 Data augmentation

To enhance the effective training and generalization capability of the deep learning model under a limited data scale, this study first implemented random flipping operations on the initial dataset, expanding the total number of training set samples to 1000 to alleviate the issue of sample scarcity. Furthermore, considering that this research focuses on the small target detection task, Mosaic data augmentation technology was specifically introduced (Dalgaty et al., 2024). This method stitches four images into one composite image, simulating complex background combinations found in real-world scenarios and allowing small targets to appear in diverse environments. This step not only increases the distribution density of small targets in the training samples but also helps to mitigate the model’s tendency to overfit to simple background correlations. On this basis, this study proceeded to use Mix-up enhancement technology to improve the model’s generalization and robustness. By linearly interpolating images and their labels, Mix-up plays a regularization role, reducing the model’s sensitivity to training samples and improving generalization performance. In MixUp, the interpolation coefficient λ was sampled from a symmetric beta distribution, λ∼Beta(α,α), and the mixed sample was generated as $x_{\text{mix}}=\lambda x_{i}+(1-\lambda )x_{j}$. The parameter α was set to 0.3 rather than 0.8. When α=0.3, the beta distribution assigns greater probability to values near 0 and 1, meaning that one source image generally remains dominant in the mixed sample. This relatively conservative mixing behavior is suitable for small pest-damage regions and weak lesion boundaries because approximately equal blending may reduce local target contrast, attenuate small feeding traces, and produce ambiguous object boundaries. Therefore, α=0.3 was adopted to retain the regularization benefit of MixUp while reducing target dilution and boundary ambiguity. This value was treated as a task-oriented training setting rather than an independently optimized methodological contribution.

**Figure 2:**
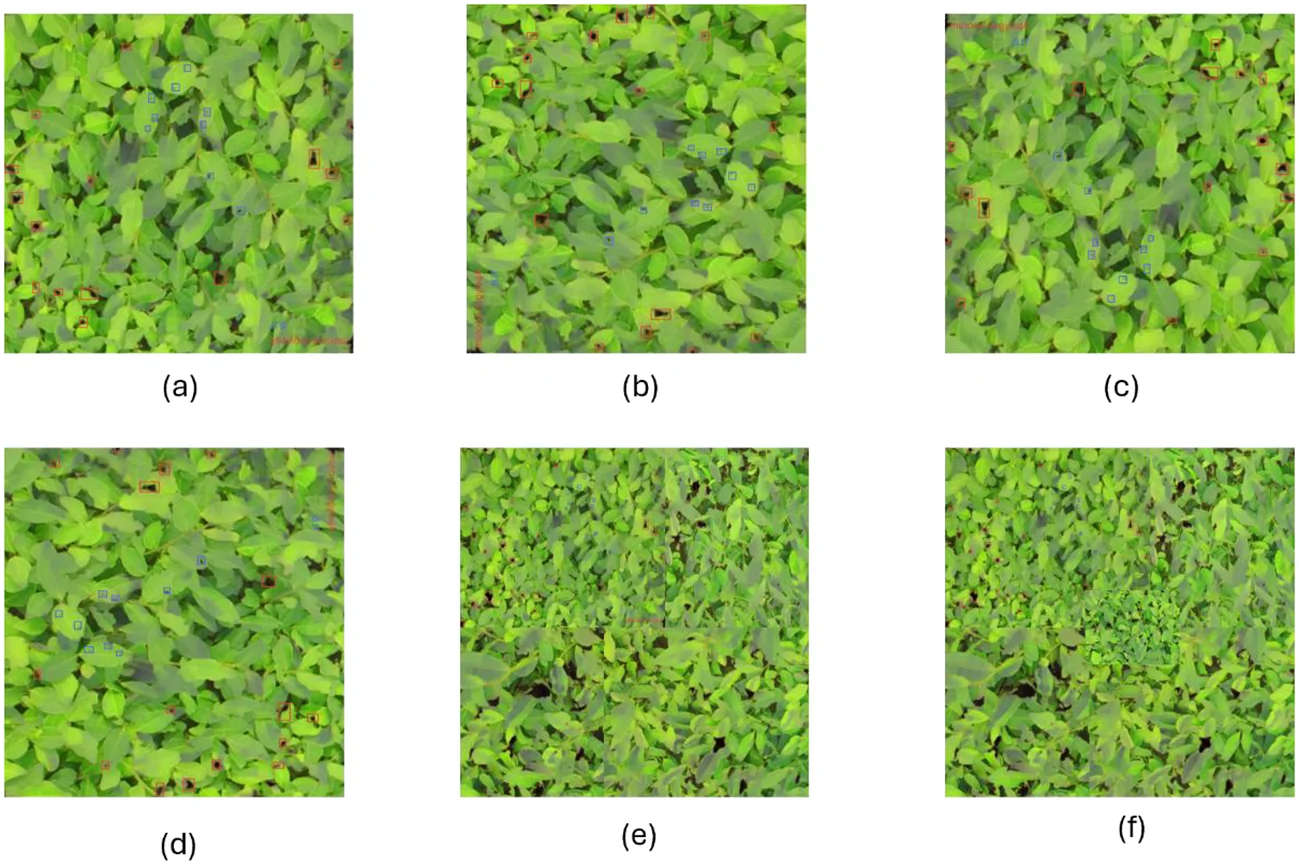
Effects of three data augmentations. **(a)** Original image, **(b)** Randomly flipped 90°, **(c)** Randomly flipped 270°, **(d)** Randomly flipped 180°, **(e)** Four images randomly stitched by Mosaic, **(f) **Effect of Mix-up applied to the original image and **(e)**.

Before data augmentation, the label counts for TLB and Apolygus lucorum were 4,071 and 3,840, respectively. After data augmentation, the label counts for *Apolygus lucorum* and TLB increased to 10,624 and 13,006, respectively. **Figure 2** shows sample examples after the three data augmentation steps were applied. The specific label counts are shown in **Table 3**.

**Table 3:** Distribution of dataset labels.

Distribution	Before data augmentation	After data augmentation
Images	445	3571
Leaf blight	4071	13006
Apolygus lucorum	3840	10624

## 3 Methods

### 3.1 TEA-LWNet

**Figure 3:**
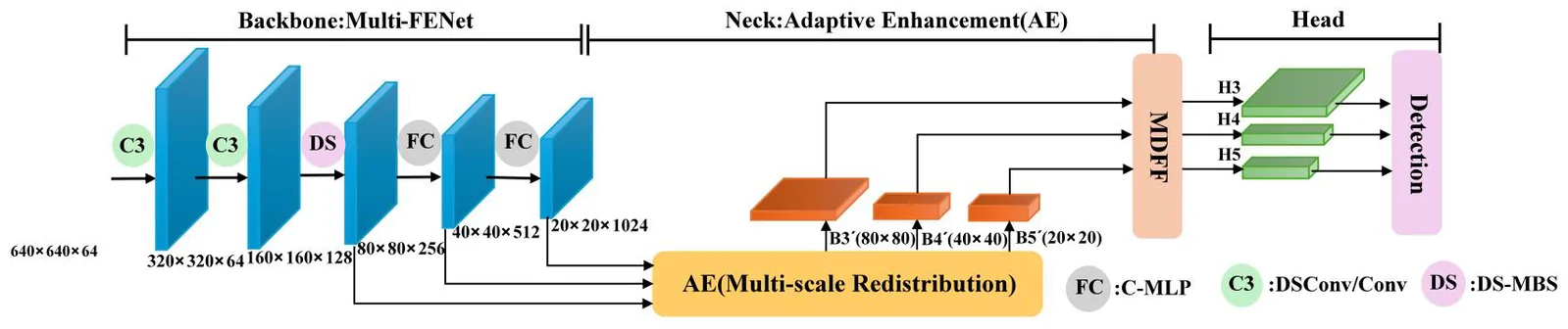
TEA-LWNet model structure diagram.

This paper proposes TEA-LWNet, a multi-scale detection framework that balances lightweight design with small-target robustness, aiming to improve the detection accuracy of Apolygus lucorum and TLB targets while maintaining low model complexity. TEA-LWNet consists of three main components: a Multi-FE backbone for hierarchical feature extraction, a neck composed of AE and MDFF for spatial–semantic enhancement and multi-scale feature fusion, and a three-branch detection head for classification and bounding box regression. The backbone primarily uses convolution and depthwise separable convolution, supplemented by linear projection, to achieve progressive downsampling and channel compression, outputting hierarchical features B3, B4, and B5. AE performs spatial alignment and semantic calibration on these multi-level features and suppresses redundancy while enhancing discriminability through high- and low-order channel decomposition and adaptive weight redistribution. Subsequently, MDFF constructs bidirectional feature fusion paths and completes cross-layer interaction through lateral connections, concatenation, and lightweight convolution, producing fused multi-scale features that are further refined into P3, P4, and P5 for the detection head. Finally, the three-scale detection head performs class prediction and bounding box regression in parallel. Benefiting from designs such as separable convolution, channel projection, and path redistribution, TEA-LWNet effectively enhances small target recall and overall detection performance in complex backgrounds while significantly reducing FLOPs and parameter count. The model structure diagram of TEA-LWNet is shown in **Figure 3**.

### 3.2 Multi-FENet

**Figure 4:**
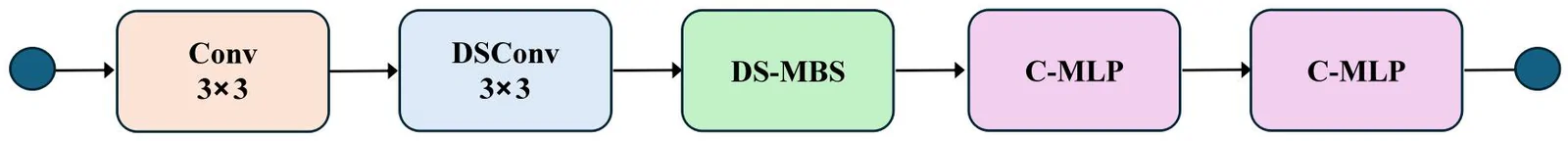
Structure of Multi-FENet.

Multi-FENet serves as the backbone’s core, adopting a progressive “feature extraction-downsampling” paradigm (**Figure 4**).

To mitigate aliasing and contour loss in small targets, the input first undergoes stride convolution for initial encoding and noise suppression. Stage-wise blocks then utilize Depthwise Separable Convolution (DSConv) (Zhang and Zhang, 2024) and the innovative Depthwise-Separable Multi-stage Bottleneck Stack (DS-MBS) to compress spatial resolution while expanding channel bandwidth. Specifically, DS-MBS employs parallel Split-MBS branches to capture multi-scale context, ensuring dual adaptation to flaky lesions and fine-grained textures.

In later stages, Multi-FENet integrates the C-MLP module to model long-range dependencies via global statistics-driven recalibration, enhancing class discrimination without self-attention’s quadratic complexity. The backbone ultimately outputs three hierarchical feature layers: detail-sensitive B3, balanced B4, and semantically condensed B5. These outputs naturally facilitate subsequent multi-scale redistribution in the AE and bidirectional fusion in the MDFF components.

### 3.3 DS-MBS

**Figure 5:**
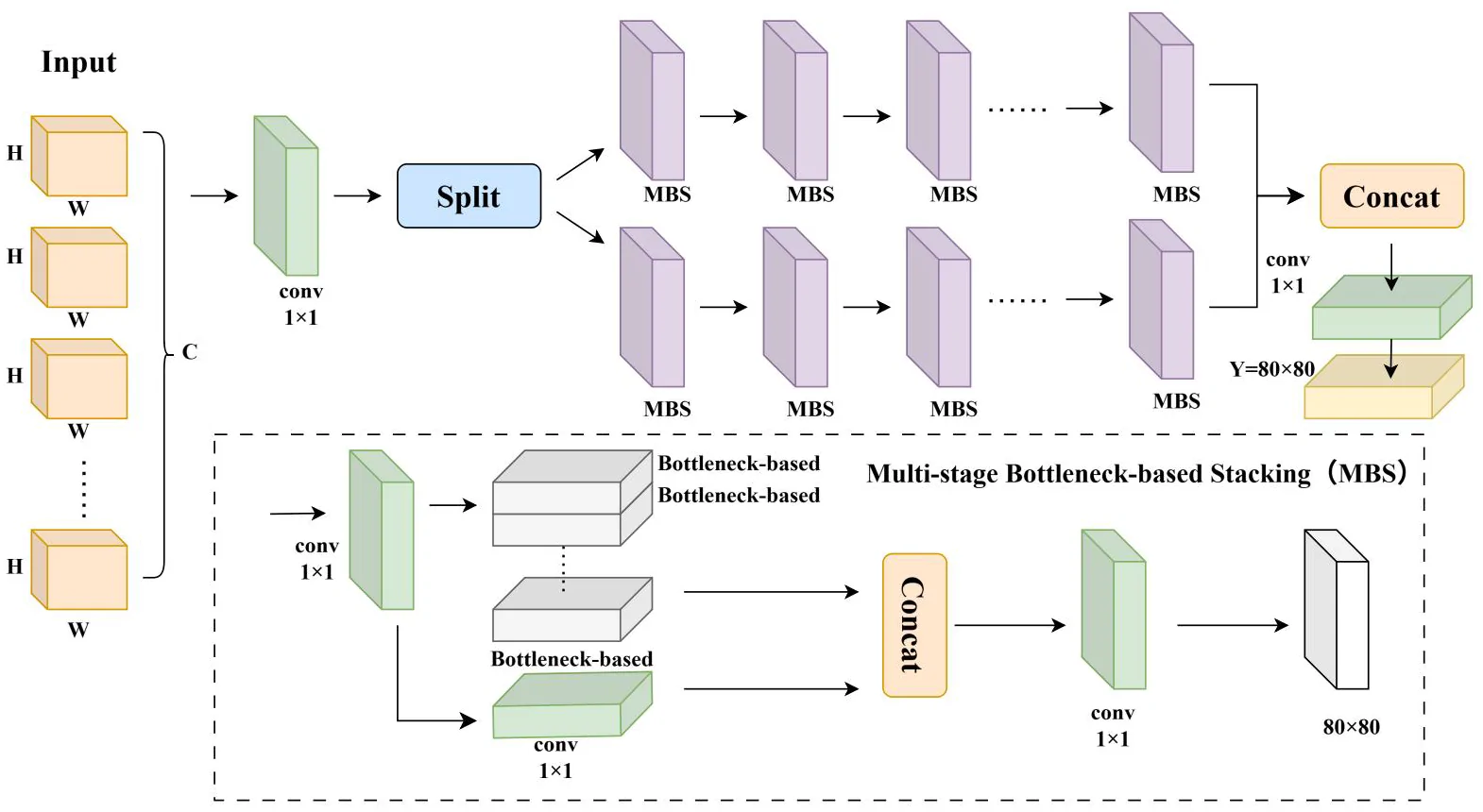
DS-MBS structure diagram.

In the initial design of the backbone network, we adopted standard convolution (Conv) as the basic component and downsampling unit (Ye et al., 2024b). However, experiments and visualization results indicated that early downsampling easily causes the loss of small target details, the decay of gradient propagation, and insufficient representation capability for scale-heterogeneous features. To alleviate the above problems, this paper designs DS-MBS as a composite unit for simultaneous downsampling and multi-scale modeling. Its structure is shown in **Figure 5**.

Given an input feature, it first obtains channel alignment through a linear projection, and is then split along the channel dimension into two branches: The first is the “high-order” path, which stacks several Multi-stage Bottleneck Stacking (MBS) modules (used to cover different receptive fields and suppress redundancy at extremely low cost, detailed in Section 3.3.1) with different kernel sizes and dilation rates, to explicitly expand the equivalent receptive field for modeling the long-range structure of flaky lesions. The second is the “low-order” path, which still uses a lightweight MBS group to preserve the high-frequency edges and textures of small-bodied targets like *Apolygus lucorum*. The outputs from the two paths are concatenated and then linearly fused using a 1×1 convolution, accompanied by a projection residual to stabilize training. The transformation of DS-MBS is defined in Equation 4:

(4) $$\begin{array}{c} Y=\phi (Conv_{1\times 1}([G_{high}(\tilde{X}),G_{low}(\tilde{X})]))+\Pi (X) \end{array}$$

Where $G_{\text{high}}$ and $G_{\text{low}}$ represent the high-order and low-order MBS series operators, respectively, Π is the identity or projection shortcut to match the stride, ϕ represents normalization and non-linearity, and Y is the 80x80 feature map. To reduce computation while ensuring spatial modeling capability, DS-MBS uniformly adopts depthwise separable convolution. The theoretical complexities of standard convolution and depthwise separable convolution are given in Equations 5 and 6, respectively:

(5) $$\begin{array}{c} F_{\text{std}}=k^{2}C_{in}C_{out}HW \end{array}$$

(6) $$\begin{array}{c} F_{\text{dw}}=k^{2}C_{in}HW+C_{in}C_{out}HW \end{array}$$

When k=3 and k=3 are moderate, $F_{\text{dw}}\ll F_{\text{std}}$. In summary, DS-MBS simultaneously achieves long-range semantic introduction, detail fidelity, and gradient stability within a single stride-based downsampling, providing a discriminative and fusible multi-scale representation for the subsequent milestone feature B3.

#### 3.3.1 MBS

In DS-MBS, we found that relying solely on single-path convolution makes it difficult to simultaneously characterize the low-frequency (Zhou et al., 2025), large-scale structure of flaky lesions and the high-frequency textures and fine-grained edge features of minute damage instances (micro-spots, point spots, necrotic boundaries) caused by *Apolygus lucorum* feeding or piercing. Furthermore, after early stride-2 downsampling, the equivalent receptive field of a single convolution is insufficient and unfriendly to small targets, while also suffering from channel redundancy and training instability. To this end, MBS (k=3) is introduced into the two parallel branches of the C3 paradigm, as illustrated in the DS-MBS structure in **Figure 5**. It progressively expands the global context in a multi-stage, low-cost manner while retaining high-frequency details. Assuming that the input has been aligned by projection, the initialization and stage-wise update of MBS within each path are defined in Equations 7 and 8, respectively:

(7) $$\begin{array}{c} {\text{H}}^{\left(0\right)}=\tilde{\text{X}} \end{array}$$

(8) $$\begin{array}{c} {\text{H}}^{\left(l+1\right)}=F_{\text{DBS}}^{\left(l\right)}({\text{H}}^{\left(l\right)}),l=0,1,2 \end{array}$$

where $F_{\text{DSB}}^{\left(l\right)}$ is the l stage bottleneck-based unit (see 3.3.2).

High-order branch: Uses increasing kernel sizes and dilation rates across stages (e.g., $k_{l}\;↑$ or $d_{l}\;↑$, with $s_{0}=2$ in the first stage and $s_{1,2}=1$ thereafter), and the equivalent receptive field and effective kernel size are computed using Equations 9 and 10, respectively:

(9) $$R_{l+1}=R_{l}+(k_{eff}^{\left(l\right)}-1)\prod_{i\leq l}s_{i}$$

(10) $$\begin{array}{c} k_{\text{eff}}^{\left(l\right)}=k_{l}+(k_{l}-1)(d_{l}-1) \end{array}$$

Low-order branch: Uses a fixed small kernel/low dilation ( $k_{l}=3,\;d_{l}=1$) to suppress aliasing and prioritize the preservation of fine-grained damage boundaries and high-frequency texture signals formed by *Apolygus lucorum* feeding or piercing, thereby enhancing the separability of small-scale damage instances. The two terminal outputs are concatenated along the channel dimension and linearly fused by a 1 × 1 convolution, as defined in Equation 11 (Chen et al., 2024):

(11) $$Z=\text{Con}{\text{v}}_{1\times 1}([{\text{H}}_{\text{high}}^{\left(3\right)},{\text{H}}_{\text{low}}^{\left(3\right)}])$$

It is then added to the projection shortcut to enable stable stride-2 downsampling. In terms of complexity, the computational cost of a single stage is approximated by Equation 12:

(12) $$\begin{array}{c} F_{\text{stage}}\approx HW(C_{in}C_{mid}+k_{l}^{2}C_{mid}+C_{mid}C_{out}) \end{array}$$

Compared to a standard 3x3 convolution, it eliminates the quadratic term related to channels, growing linearly with the number of channels while ensuring spatial modeling; when combined with lightweight attention, it can further suppress redundant channels and enhance discriminative dimensions. Thus, MBS achieves “progressive receptive-field expansion, detail preservation, and stable residual training” without significantly increasing computation, laying the foundation for DS-MBS to simultaneously obtain strong semantics and fine-grained details within a single downsampling.

#### 3.3.2 Bottleneck-based unit

**Figure 6:**
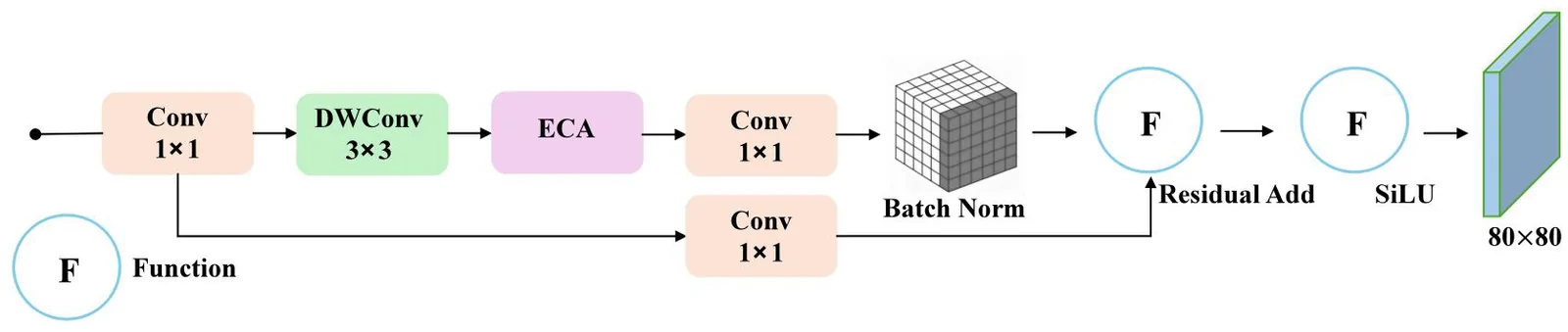
Bottleneck-based structure diagram.

After early downsampling, detail loss and gradient decay easily occur; directly using multiple convolutions causes inefficient channel mixing and excessive computational load. Meanwhile, the discriminative dimensions for tea leaf lesions are sparse and uneven. Based on this, we draw inspiration from Bottleneck (Shang et al., 2024) and design a lightweight Bottleneck-based unit as the atomic unit for MBS. First, it performs channel expansion, then sequentially executes channel-wise spatial modeling, followed by lightweight feature recalibration, and then undergoes channel projection to compress dimensions. Finally, it combines with the original feature through residual fusion to achieve spatial-channel decoupling and stable training with minimal overhead (Hu et al., 2023). The specific structure of the Bottleneck-based unit is shown in **Figure 6**.

First, to address the problem of limited channel expression after early downsampling, a 1 × 1 convolution is applied to the input U for channel expansion and linear mixing, and the expanded feature P is obtained using Equation 13:

(13) $$\begin{array}{c} P=\text{BN}(\text{Con}{\text{v}}_{1\times 1}(\text{U};\;C_{\;mid}=rC_{\;in})) \end{array}$$

to enhance the representation dimensionality and provide a sufficient channel basis for subsequent channel-wise modeling. Secondly, to obtain an effective receptive field at a lightweight cost while also accommodating downsampling, channel-wise spatial modeling is completed using DWConv, as defined in Equation 14:

(14) $$\begin{array}{c} V=\text{BN}(\text{DWCon}{\text{v}}_{k\times k}^{\left(s,d\right)}(\text{P})) \end{array}$$

Subsequently, the fully-connected layer-free ECA attention is introduced for channel recalibration, as expressed in Equation 15:

(15) $$\begin{array}{c} \alpha =\sigma (\text{Conv}1{\text{D}}_{k_{\;eca}}(\text{GAP}(\text{v}))) \end{array}$$

The weighted features are obtained using Equation 16:

(16) $$\begin{array}{c} {\text{V}}^{\prime }=V⊙\alpha \end{array}$$

and uses a 1×1 convolutional projection to compress and align the interface. Finally, to suppress gradient decay and mitigate information collapse caused by downsampling, a shortcut path consistent with the main branch stride is introduced for residual fusion, and a smooth non-linearity is applied to generate the output feature, as shown in Equation 17:

(17) $$\begin{array}{c} \hat{\text{Y}}=SiLU(\text{Con}{\text{v}}_{1\times 1}({\text{V}}^{\prime })+{\text{Π}}_{s}(\text{U})) \end{array}$$

Through this integrated process of channel mixing, spatial modeling, lightweight recalibration, and residual fusion, the unit achieves detail preservation, semantic enhancement, and training stability.

#### 3.3.3 C-MLP

**Figure 7:**
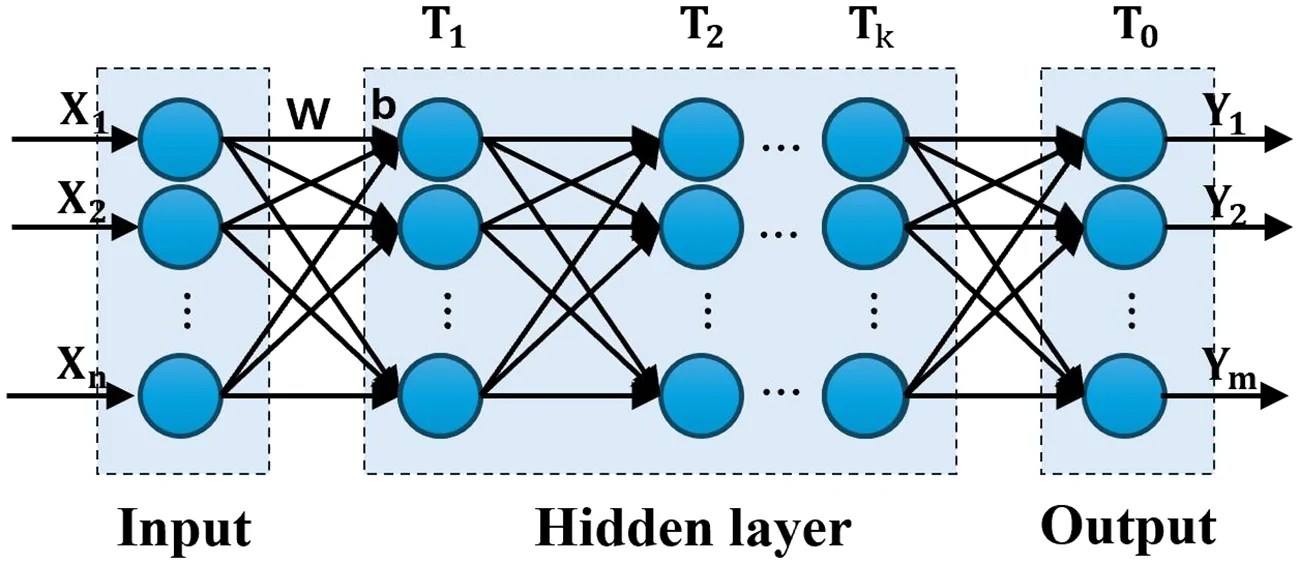
MLP structure diagram.

In tea plantation UAV imagery, pure convolutional networks often struggle to detect small targets—such as spots or insect holes—because their local receptive fields may fail to preserve fine details under motion blur and complex backgrounds. Expanding the field of view in such networks leads to rapidly escalating computational costs. Conversely, while multi-layer perceptrons (MLPs) offer advantages in cross-channel modeling and long-range dependencies (Tolstikhin et al., 2021), they suffer from a lack of spatial perception, high memory overhead, and a risk of overfitting in small-sample conditions. The standard MLP structure is illustrated in **Figure 7**.

**Figure 8:**
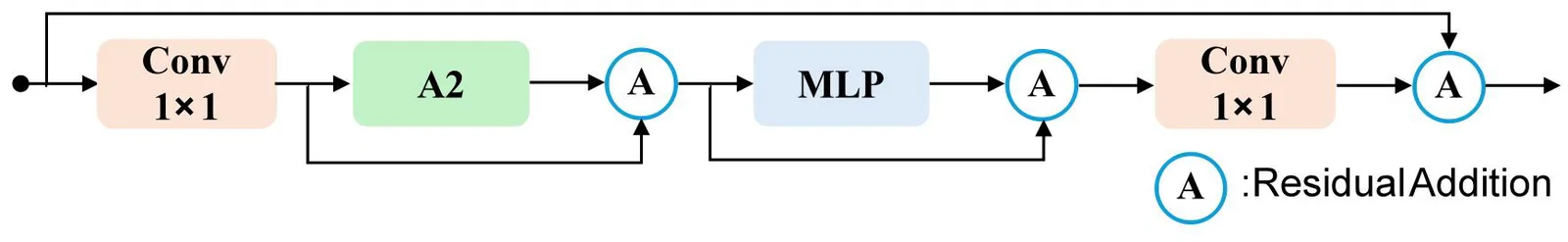
C-MLP structure diagram.

To overcome these challenges, this paper introduces the C-MLP module at key network positions to balance local texture extraction and cross-channel interaction with minimal computational overhead. Its specific structure is shown in **Figure 8**.

The module processes features through two parallel paths: one path uses depthwise separable convolution to encode neighborhood intensity and edge textures, strengthening lesion details; the other path utilizes a channel MLP to mix inter-channel correlations and long-range dependencies, adapting to multispectral statistical differences. The fused outputs are compressed via 1x1 projection and calibrated by lightweight channel attention to suppress background interference. Primarily composed of pointwise and depthwise convolutions, this structure maintains relatively low computational overhead and is potentially applicable to resource-constrained UAV image-processing scenarios (Lu et al., 2024). The introduction of C-MLP effectively amplifies fine-grained structures and improves small-target recall.

### 3.4 AE

**Figure 9:**
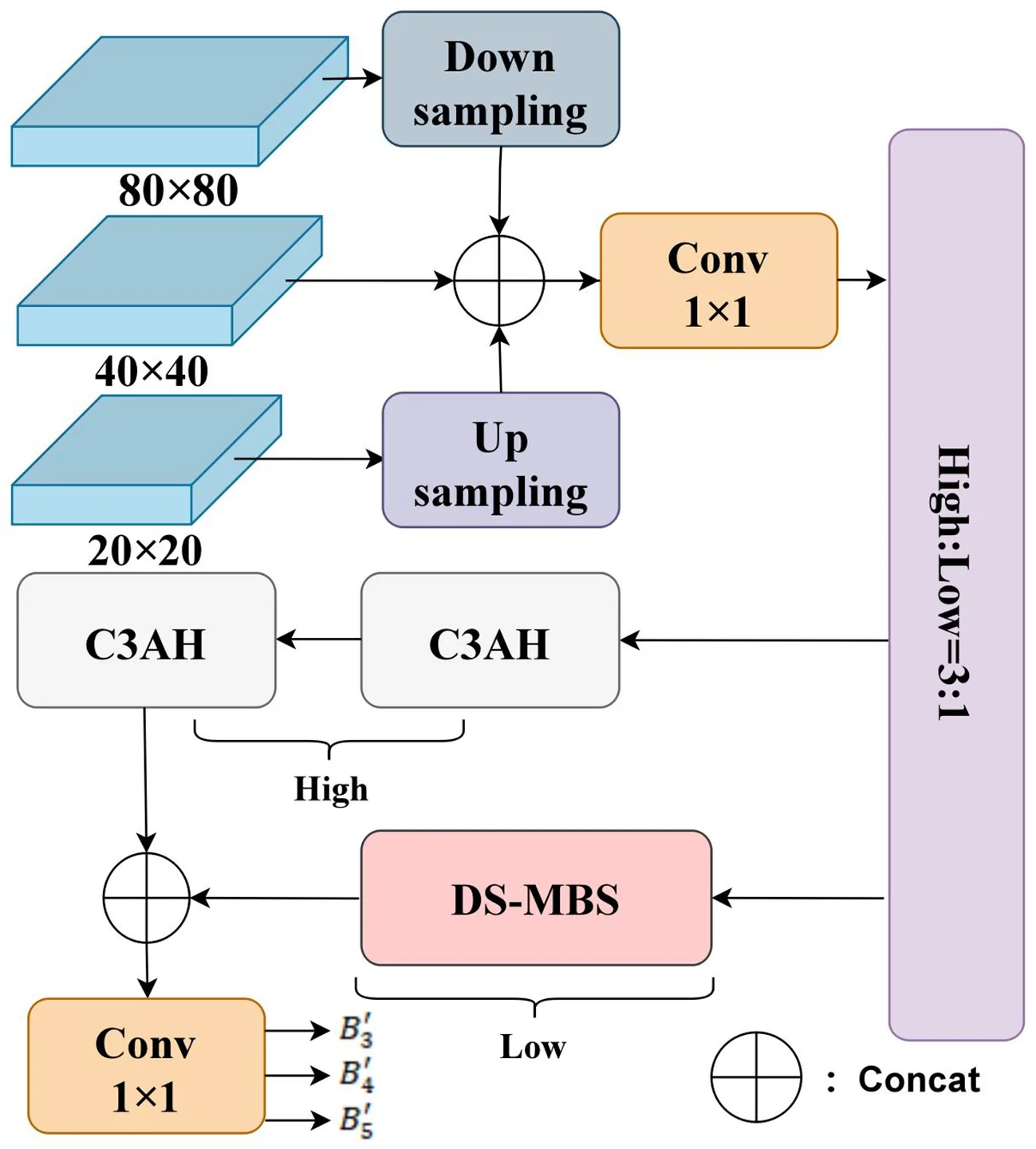
AE structure diagram.

When designing the neck component, traditional FPN and PAFPN fusion methods often face three critical limitations: difficulty in semantic alignment across different levels leading to scale aliasing; significant variance in channel statistics that amplifies redundant information; and the limited ability of a single fusion path to capture both large-scale lesions and fine-grained damage boundaries caused by Apolygus lucorum feeding (Pan et al., 2024). To address these, this paper introduces the AE module. The core philosophy is to first unify the spatial scale and perform lightweight linear fusion with channel rearrangement, followed by a branch-wise decomposition strategy using asymmetric high- and low-order branches. These branches independently process long-range semantics and high-frequency details before reconverging into a more separable multi-scale representation. The structural diagram is shown in **Figure 9**.

The AE module execution follows these steps:

First, spatial alignment and multi-source aggregation are performed by unifying the three scale features from the backbone to a common resolution. This is achieved through learnable depthwise separable convolutions for downsampling and nearest-neighbor interpolation for upsampling, thereby avoiding sampling artifacts and semantic misalignment typical of direct cross-layer addition. Subsequently, a pointwise convolution completes linear mixing and channel compression.

**Figure 10:**
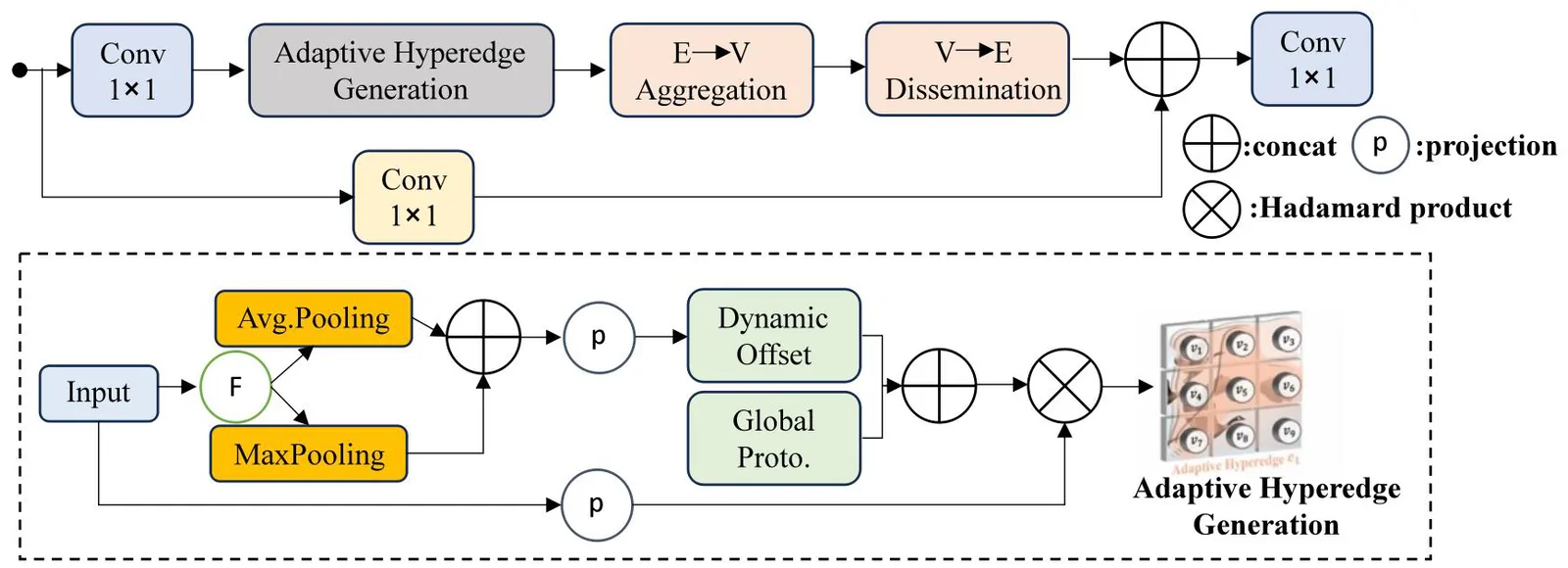
Structure of the C3AH module. Spatial features are treated as vertices, dynamic prototypes define the adaptive hyperedges, and H denotes the soft association matrix. V → E and E → V represent vertex-to-hyperedge aggregation and hyperedge-to-vertex propagation, respectively.

The features are then split into two groups along the channel dimension at a 3:1 ratio. The high-order branch (accounting for 3/4) is dedicated to capturing global and non-local information via the C3AH module (Lei et al., 2025), illustrated in **Figure 10**.

To overcome the local receptive-field limitation of CNNs, C3AH constructs a dynamic hypergraph from the high-order feature branch. Given an input feature map $X\in {\mathbb{R}}^{C\times h\times w}$, its spatial dimensions are flattened into N=h×w vertices, with each vertex representing a spatial feature vector. A set of M learnable global prototypes is associated with input-dependent dynamic offsets to generate M adaptive hyperedges. In this study, M=8. The soft association matrix $\text{H}\in {\left[0,1\right]}^{N\times M}$ represents the membership relationships between spatial features and adaptive hyperedges. Information is then exchanged through vertex-to-hyperedge aggregation and hyperedge-to-vertex propagation:

Hyperedge-to-vertex propagation (E→V), which propagates the aggregated hyperedge information back to the vertex features:

(18) $$v_{i}^{'}=\sigma (\sum_{e_{j}\in E}H(i,j)\cdot \phi (e_{j}))$$

Vertex-to-hyperedge aggregation (V→E), which aggregates the features of the participating vertices into each hyperedge:

(19) $$e_{j}^{'}=\sigma (\sum_{v_{i}\in Y}H(i,j)\cdot \psi (v_{i}))$$

In Equations 18, 19, ${\text{H}}_{ij}$ denotes the soft membership weight between vertex $v_{i}$ and hyperedge $e_{j}$. A larger value of ${\text{H}}_{ij}$ indicates that the spatial feature represented by $v_{i}$ contributes more strongly to hyperedge $e_{j}$. The association matrix is generated by applying a softmax operation to the scaled similarity between the projected vertex features and the dynamic hyperedge prototypes. Each row of H therefore describes the participation of one spatial vertex in the M hyperedges. Here, ψ(·) and ϕ(·) denote learnable feature projections, and σ(·) denotes the nonlinear activation function. The actual message-passing cycle is executed in the order of V→E followed by E→V. Equation 19 first aggregates semantically related vertex features into each hyperedge. Equation 18 then propagates the resulting hyperedge information back to the participating vertices. For simplicity, the updated hyperedge representation obtained from Equation 19 is denoted by $e_{j}$ when it is used in Equation 18. A residual connection is retained during vertex updating to preserve the original spatial information. The simplified computational procedure is summarized in **Table 4**.

**Table 4:** Simplified pseudocode for adaptive hypergraph construction and message passing in C3AH.

Input: high-order feature X and number of hyperedges M
V = Flatten(X) # N spatial vertices context = Concat(GlobalAveragePool(V), GlobalMaxPool(V)) P = GlobalPrototypes + Offset(context) # M dynamic prototypes H = Softmax(Similarity(Project(V), P)) # N × M matrix E = Normalize(H^T × Project(V)) # V → E aggregation V_new = V + Normalize(H × Project(E)) # E → V propagation Y = Reshape(V_new) Output: correlation-enhanced feature Y

For example, spatially separated regions belonging to the same irregular TLB lesion may obtain high membership weights for the same dynamic hyperedge because they share similar necrotic texture, gradual color transition, and boundary characteristics. During V→E aggregation, these spatially separated features are collected by the shared hyperedge. During E→V propagation, the aggregated lesion context is returned to each participating vertex. Consequently, a weak or partially occluded lesion region can be reinforced by other semantically consistent regions even when they are not spatially adjacent. Meanwhile, the parallel DS-MBS branch preserves local boundaries and small punctate feeding traces caused by Apolygus lucorum.

Standard dense self-attention constructs an N×N affinity matrix and models pairwise relationships between all spatial tokens, resulting in approximately $O(N^{2}d)$ computational complexity and $O(N^{2})$ memory consumption. In contrast, C3AH constructs an N×M association matrix between N spatial vertices and M dynamic hyperedges. A hyperedge can simultaneously connect multiple spatially separated but semantically consistent vertices, thereby representing group-wise high-order relationships. Its association construction and message-passing operations require approximately O(NMd) computation and O(NM) memory. Since M≪N, the complexity is approximately linear with respect to the number of spatial vertices.

At an input resolution of 640×640, the complete TEA-LWNet required 7.63 GFLOPs. When C3AH was replaced with a shape-matched 1×1 projection, the computational cost decreased to 7.41 GFLOPs. Therefore, C3AH introduced 0.22 GFLOPs, accounting for approximately 2.88% of the total model computation. This result indicates that C3AH provides group-wise non-local information interaction with a relatively limited computational overhead.

The low-order branch (accounting for 1/4) is fed into the DS-MBS unit described previously. It utilizes small kernels and lightweight channel attention to suppress aliasing while preserving fine-grained boundaries and textures.

Finally, the enhanced results from both branches are concatenated and linearly integrated. The AE module generates three spatially and semantically enhanced features, denoted as A3, A4, and A5, for subsequent cross-scale interaction. By combining group-wise high-order contextual modeling in C3AH with local-detail preservation in DS-MBS, AE improves semantic consistency and boundary separability, providing more informative multi-scale features for the subsequent MDFF module.

### 3.5 MDFF

**Figure 11:**
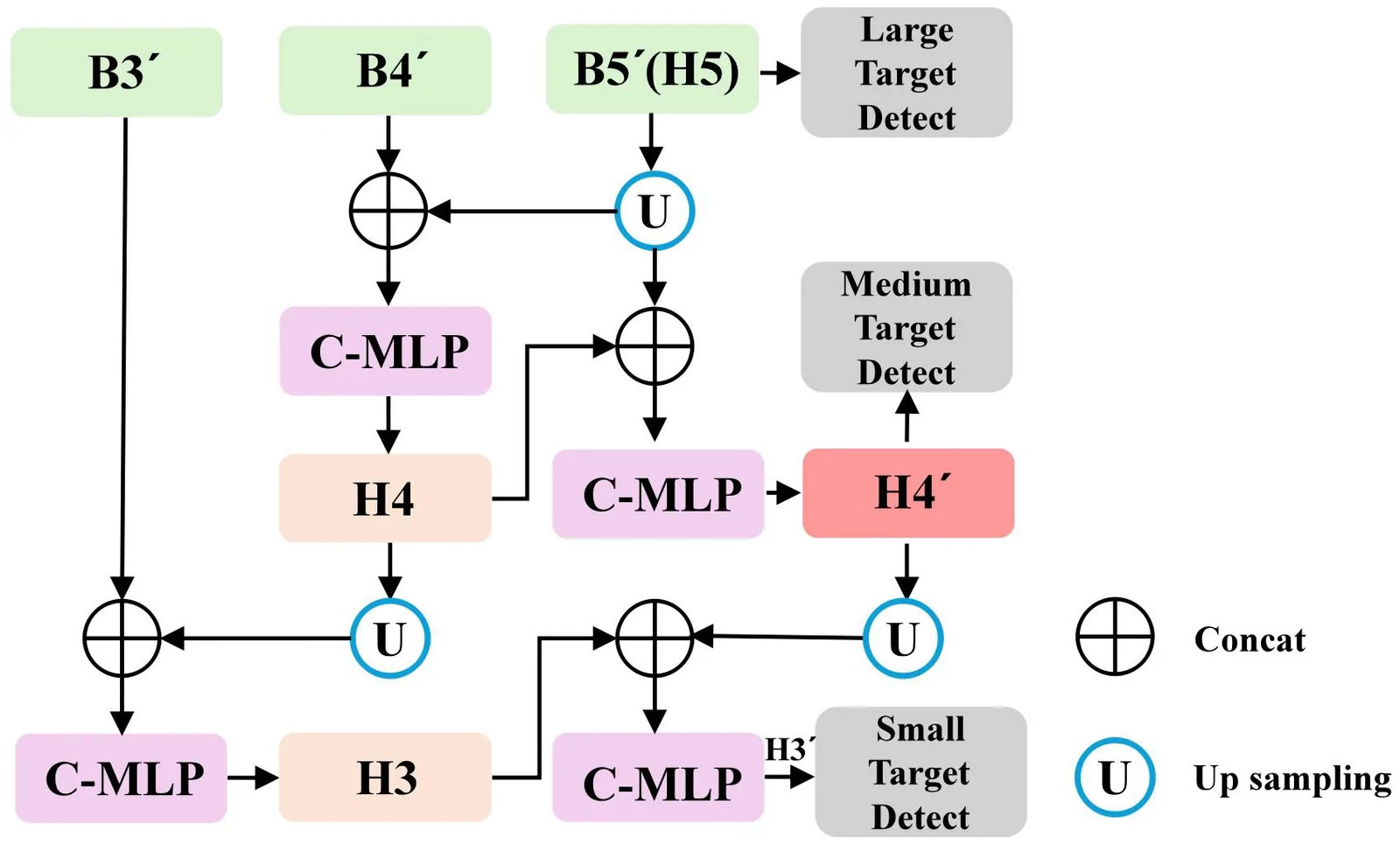
MDFF structure diagram.

To address the problem of small targets being easily missed in detection, the core design of MDFF is a continuous two-stage top-down path with upsampling. This path injects strong top-level semantics step-by-step into higher-resolution layers, thereby enhancing the separability and recall of small targets without significantly increasing computational power. Its structure is shown in **Figure 11**.

The MDFF first performs lateral alignment on the features from each layer to eliminate channel and statistical discrepancies, as formulated in Equation 20:

(20) $$L_{l}=SiLU\;(\text{BN}(\text{Con}{\text{V}}_{1\times 1}(B_{l}^{'};\;C_{\text{fuse}}))),\;\;\ell \in \{3,4,5\}$$

and retains $H_{5}=L_{5}$ as the strong semantic branch for large targets. Subsequently, two upsampling steps are executed: The first step is to upsample $H_{5}$ to the scale of $L_{4}$ to enhance the middle layer (denoted as ${\tilde{H}}_{4}$, the second step is to further upsample the refined $H_{4}$ to the scale of $L_{3}$ to enhance the small target layer (denoted as ${\tilde{H}}_{3}$). To avoid the domain conflicts, semantic flooding, and upsampling aliasing introduced by “direct addition” after the two upsampling steps, MDFF introduces Gated-Fusion (GF) at each cross-layer fusion point to perform a controlled injection of “select first (Wei et al., 2024), then reconstruct,” as defined in Equation 21:

(21) $${\tilde{H}}_{l}=Upsample(H_{l+1}),\;\;F_{l}=GF(L_{l},{\tilde{H}}_{l}),\;\;H_{l}={\text{Φ}}_{\text{C}-\text{MLP}}(F_{l}),\;\;\ell \in \{4,3\}$$

where Φ denotes the lightweight refinement block based on channel reconstruction and interface projection. Nearest-neighbor interpolation is adopted for upsampling. The channel and spatial gate coefficients and the resulting fused feature are defined in Equations 22 and 23, respectively:

(22) $$\alpha =\sigma \;(W_{\alpha }\text{ GAP}(\hat{H})),\;\;\beta =\sigma \;(W_{\beta }\text{ GAP}(L)),\;\;M=\sigma \;(\text{Con}{\text{v}}_{1\times 1}([L,\hat{H}]))$$

(23) $$\begin{array}{c} F=\beta ⊙L+(\alpha ⊙M)⊙\hat{H} \end{array}$$

On this basis, considering that the medium scale is the most sensitive to context, MDFF retains a top-level reflow that is controllably injected via GF to obtain H′4, as defined in Equation 24:

(24) $$\begin{array}{c} H_{4}^{'}={\text{Φ}}_{\text{C}-\text{MLP}}\;(\text{GF}(H_{4},\text{Upsample}(H_{5}))) \end{array}$$

The two-stage upsampling and GF-controlled fusion strategy improves small- and medium-scale target detection. The high-resolution layer benefits from the continuous injection of global and mid-level semantic information, while GF suppresses task-irrelevant upstream responses in both channel and spatial dimensions. This controlled fusion alleviates semantic flooding, background diffusion, and boundary fragmentation. At the same time, the top-level feature retains strong semantic representation for larger targets. Since GF has a computational complexity comparable to lightweight convolution and C-MLP, MDFF improves multi-scale feature interaction while maintaining a lightweight structure.

### 3.6 Loss function: SAB-loss

For the three-scale anchor-free detection head at P3, P4, and P5, we adopt a compound training objective consisting of Complete Intersection over Union (CIoU) loss, Distributed Focal Loss (DFL), and binary cross-entropy (BCE) loss with label smoothing. CIoU is used for bounding-box regression by jointly considering the overlap area, center-point distance, and aspect-ratio consistency between the predicted and ground-truth boxes. DFL provides distributional supervision for bounding-box boundaries, while BCE supervises category prediction. These established loss components have been widely applied in modern object detectors (Zheng et al., 2020; Li et al., 2020). In this study, they are combined to provide complementary supervision for bounding-box localization, boundary distribution learning, and category prediction in the anchor-free detection head.

Let l∈{3,4,5} denote the detection level corresponding to P3, P4, and P5, $P_{l}$ denote the set of positive samples assigned by task-aligned label assignment (TAL), and $\omega_{l}$ denote the weight of each detection level. The overall training objective is defined in Equation 25:

(25) $$\begin{array}{c} L_{\text{total}}=L_{\text{IoU}}+\lambda_{\text{DFL}}L_{\text{DFL}}+\lambda_{\text{cls}}L_{\text{cls}} \end{array}$$

For each positive sample  i, the localization loss is calculated between the predicted bounding box and its corresponding ground-truth box:

(26) $$\begin{array}{c} L_{\text{IoU}}=\sum_{l}\omega_{l}\;\sum_{i\in P_{l}}(1-\text{IoU}({\hat{\text{b}}}_{i},{\text{b}}_{i})+\text{Δ}({\text{b}}_{i},{\text{b}}_{i})) \end{array}$$

In Equation 26, the IoU term measures the overlap between the predicted and ground-truth boxes, while Δ(·) accounts for center-point distance and aspect-ratio consistency following the CIoU formulation (Zheng et al., 2020). This term provides normalized geometric supervision for bounding-box regression across different target scales.

Following Li et al. (2020), DFL represents the four bounding-box boundaries as discrete probability distributions. For each boundary c∈{l,t,r,b}, the continuous target $y_{i,c}$ is represented using its two adjacent discrete bins, and the corresponding predicted distribution is denoted by ${\hat{\text{q}}}_{i,c}$. The original DFL formulation is retained in Equation 27:

(27) $$\begin{array}{c} L_{\text{DFL}}=\sum_{l}\omega_{l}\sum_{i\in P_{l}}\sum_{c}\text{DFL }({\hat{\text{q}}}_{i,c},\;y_{i,c}) \end{array}$$

DFL provides distributional supervision around the ground-truth boundary and is used as an auxiliary regression term for refining the predicted bounding-box locations.

For robust classification, BCEWithLogits with label smoothing is used (Yang et al., 2018): Let the class logits be ${\hat{\text{p}}}_{i}\in {\mathbb{R}}^{C}$, the smoothed label ${\text{p}}^{\text{ls}}=(1-\epsilon )\text{p}+\epsilon /C$ ( ϵ∈[0.05,0.1]), The classification loss is defined in Equation 28:

(28) $$\begin{array}{c} L_{\text{cls}}=\sum_{l}\omega_{l}\;\sum_{i\in P_{l}}\sum_{k=1}^{C}\text{BCE}({\hat{p}}_{i,k},\;p_{i,k}^{\text{ls}}) \end{array}$$

Without increasing head complexity, it improves score calibration and robustness to annotation noise and class imbalance.

### 3.7 Model training and evaluation

#### 3.7.1 Training environment and parameters

Model training was performed for 301 epochs using an input resolution of 640×640 and a mini-batch size of 4. Gradients were accumulated over 16 consecutive mini-batches, corresponding to an equivalent batch size of 64. AdamW was used as the optimizer, with $\beta_{1}=0.937$, $\beta_{2}=0.999$, $\in =1\times 10^{-8}$, and a weight decay of $5\times 10^{-4}$. The initial learning rate was set to 0.01, and the minimum learning rate was set to 0.001.

A linear warm-up strategy was applied during the first 3 epochs. Because each epoch contained 1,000 training images and the mini-batch size was 4, the warm-up stage comprised 750 mini-batch iterations. After warm-up, the learning rate was adjusted from 0.01 to 0.001 using a cosine-annealing schedule. Exponential Moving Average was enabled with a maximum decay rate of 0.9999. Gradient clipping was applied with a maximum norm of 10.0 to reduce the risk of unstable gradient updates. Automatic mixed-precision training with FP16 computation was also enabled to reduce GPU memory consumption and improve training efficiency. The hardware environment, optimizer settings, learning-rate schedule, warm-up configuration, regularization parameters, and numerical-precision settings used in all experiments are summarized in **Table 5**.

**Table 5:** Configuration of the experiment.

Item	Simulated configuration
Hardware and software	Windows 11; Python 3.8; CUDA 12.2; NVIDIA GeForce RTX 4090 (24 GB); Intel Core i9-14900KF
Input and training duration	Input size: 640 × 640; epochs: 301; batch size: 4; gradient accumulation: 16 steps; equivalent batch size: 64
Optimizer	AdamW; $\beta_{1}=0.937$; $\beta_{2}=0.999$; $\in =1\times 10^{-8}$; weight decay: $5\times 10^{-4}$
Learning-rate schedule	Initial LR: 0.01; minimum LR: 0.001; cosine annealing
Warm-up	3 epochs; 750 mini-batch iterations
Exponential Moving Average	Enabled; maximum decay: 0.9999
Gradient clipping	Enabled; maximum gradient norm: 10.0
Numerical precision	Automatic mixed-precision training enabled; FP16
Data augmentation	Random flipping; Mosaic enabled; MixUp with α=0.3

#### 3.7.2 Evaluation metrics

For the tea pest and disease object detection scenario, the model’s performance is evaluated using a matching rule based on Intersection over Union (Zhang and Zhang, 2024): a prediction box is considered a correct detection if its overlap with a ground-truth box of the same class reaches a set threshold. Two types of threshold settings are reported to cover different application contexts: AP@0.5 under a fixed threshold of 0.5, and the multi-threshold average mAP@0.5:0.95, which covers thresholds from 0.50 to 0.95 with a step of 0.05. Precision is used to characterize the ability to control false positives, Recall reflects the coverage of true targets, and F1 measures the balance between the two in a single value; Average Precision (AP) is accumulated from the Precision-Recall curve, and mAP, averaged across categories and thresholds, can robustly reflect the overall detection quality. Params represent the number of learnable weights, reflecting model capacity. It is independent of input resolution but affects VRAM and the risk of overfitting. GFLOPs characterizes the computational scale of a single inference, increasing with the rise in feature map resolution and channel count. It is related to speed but constrained by operators and memory access. Size is the disk space occupied by the weights, influenced by precision and serialization.

All metrics are calculated under unified data partitions, input resolutions, and post-processing strategies to ensure horizontal comparability between different models or ablation schemes. Precision and Recall are defined in Equation 29; F1-score, AP, and mAP are defined in Equations 30–32, respectively:

(29) $$P=\frac{TP}{TP+FP},\;\;R=\frac{TP}{TP+FN}$$

(30) $$F1=\frac{2PR}{P+R}$$

(31) $$AP=\int_{0}^{1}P(R)\;dR$$

(32) $$mAP=\frac{1}{N}\sum_{i=1}^{N}AP_{i}$$

## 4 Results

### 4.1 Training

**Figure 12:**
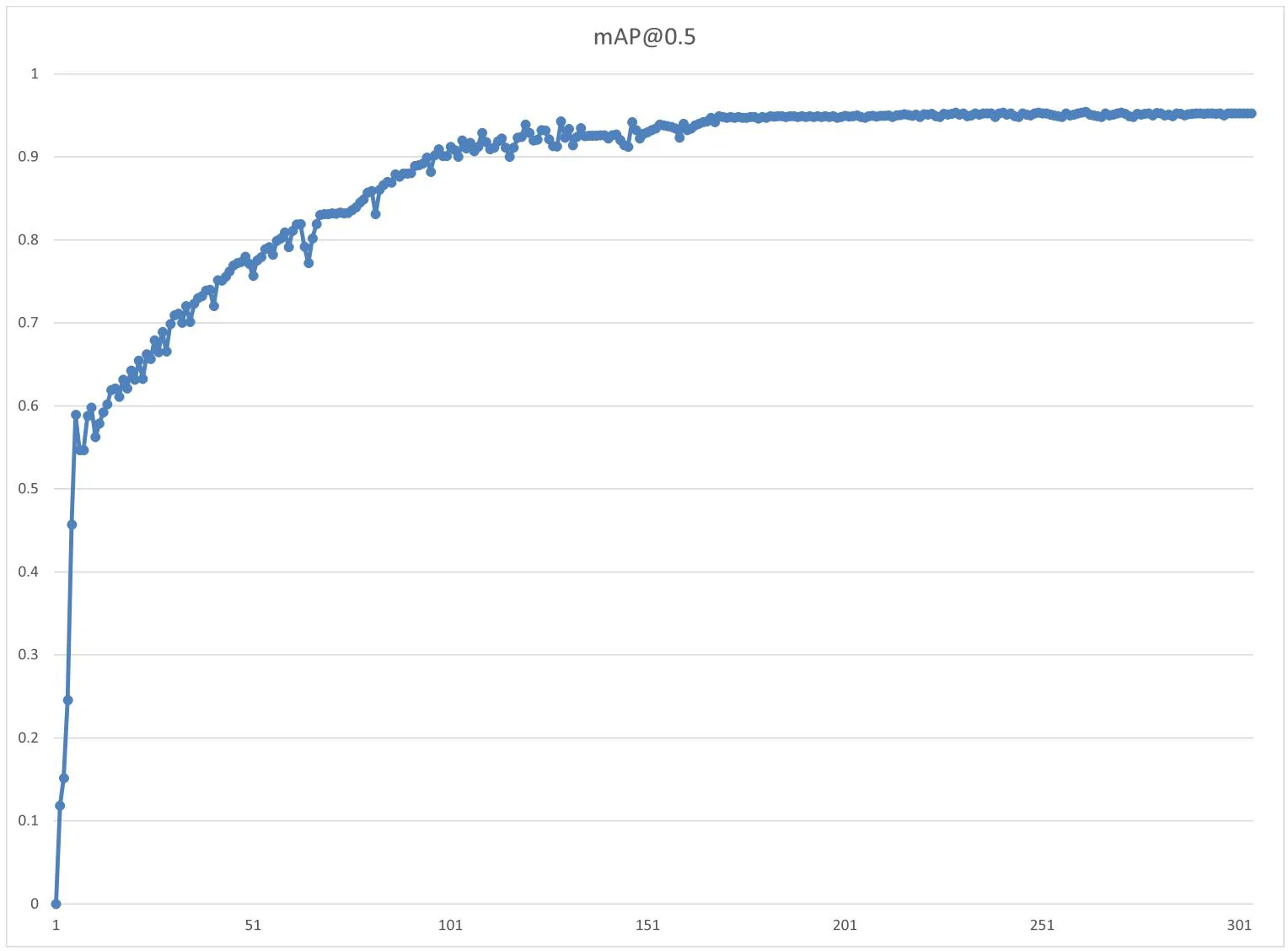
Variation of mAP@0.5 during training over 301 epochs.

**Figure 13:**
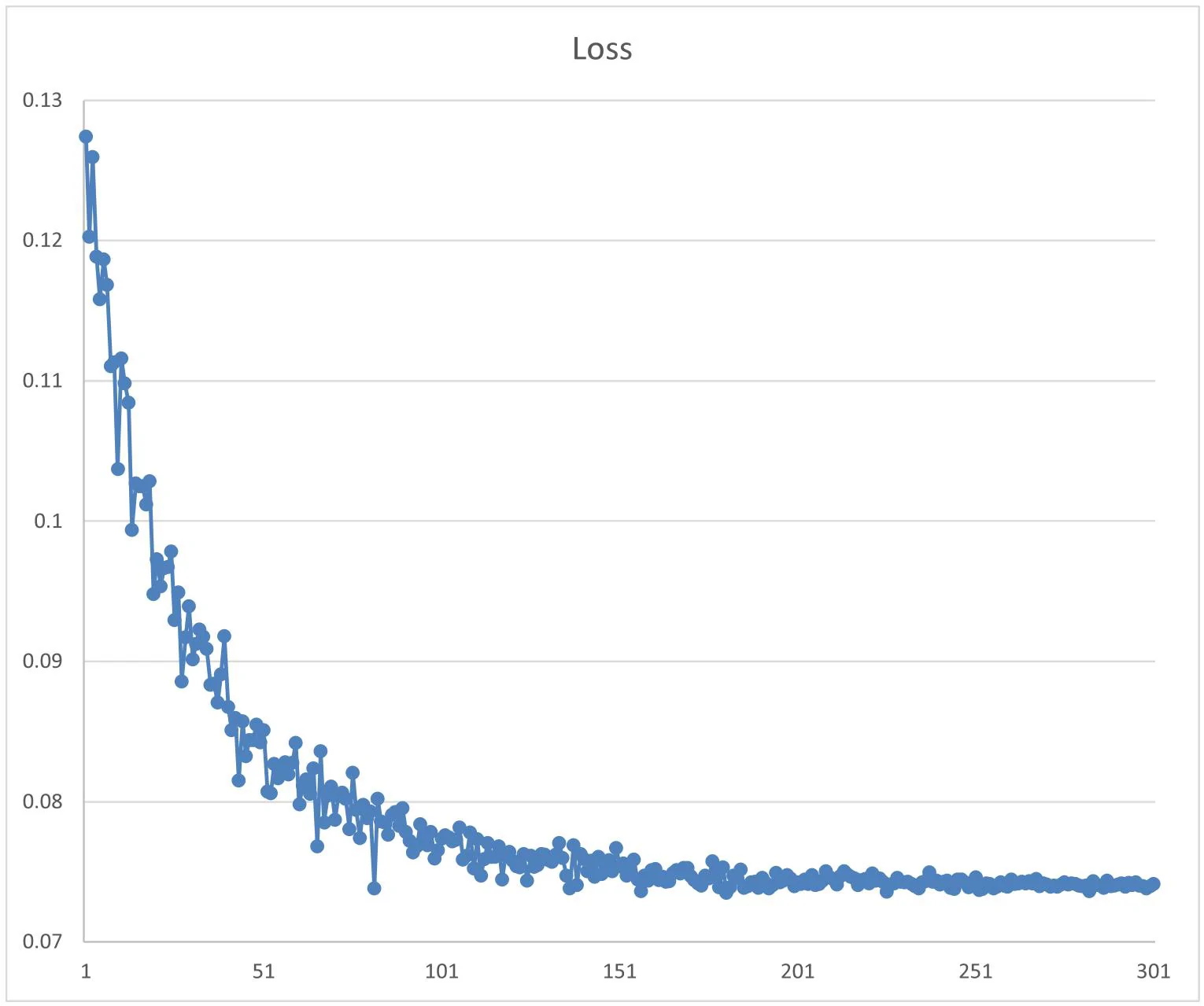
Training loss trajectory.

All models were trained using the unified environment and hyperparameter configuration reported in **Table 5**. Multi-angle 640×640 RGB-NIR fused images were used as model inputs, and the augmentation strategies described in Section 2.2 were applied only to the training set. The AdamW optimizer, 750-iteration linear warm-up, cosine-annealing learning-rate schedule, Exponential Moving Average, gradient clipping, gradient accumulation, and FP16 mixed-precision training were applied consistently across the comparative and ablation experiments. Each model was trained for 301 epochs. The corresponding mAP@0.5 and training-loss curves are presented in **Figures 12**, **13**, respectively.

**Figure 12** shows the variation of mAP@0.5 during the 301 training epochs. The mAP@0.5 increased rapidly during the early training stage and gradually stabilized after approximately 150 epochs, fluctuating between 0.93 and 0.95. This indicates that the model achieved stable validation performance. **Figure 13** shows the training loss trajectory during model optimization. The loss decreased steadily and tended to stabilize in the later epochs, indicating stable optimization without obvious divergence. Finally, under a unified inference pipeline with a 640×640input size and fixed confidence and NMS thresholds, the model achieved an mAP@0.5 of 93.48% on the test set. These results indicate that the RGB–NIR input and lightweight network structure adopted in this paper provide stable learning capability and generalization performance in the tea plantation small-target detection scenario.

### 4.2 Comparative experiments with different models

To evaluate the effectiveness of the proposed TEA-LWNet framework under a comparable computational budget, this experiment selected several representative lightweight object detectors for comparison, including YOLOv8n, YOLOv10n, YOLOv11n, MobileNet-SSD, DEMNet, and the proposed TEA-LWNet. These models were chosen because their parameter counts and GFLOPs are closer to those of TEA-LWNet than large two-stage detectors or restoration-based pipelines. All models were trained and evaluated using the same RGB-NIR fused input, data partition, input resolution, augmentation strategy, and evaluation metrics to reduce the influence of training settings and model-capacity differences. The quantitative results are shown in **Table 6**.

**Table 6:** Comparison with lightweight object detectors under similar computational budgets.

Model	P/%	R/%	mAP@0.5/%	GFLOPs	Size/MB	Params/M
YOLOv10n	83.5	78.6	86.40	6.72	10.8	2.31
YOLOv11n	85.7	80.4	88.20	6.48	10.5	2.58
YOLOv8	84.2	72.1	81.9	8.858	12.2	3.157
MobileNet-SSD	78.6	75.2	82.60	3.12	13.4	4.28
DEMNet	86.3	82.7	89.60	9.24	16.8	3.74
TEA-LWNet	88.6	89.4	93.48	7.63	13.6	1.64

As shown in **Table 6**, TEA-LWNet achieved the highest mAP@0.5 of 93.48% among the compared lightweight detectors, while maintaining only 1.64M parameters and 7.63 GFLOPs. Compared with YOLOv8n, TEA-LWNet improved mAP@0.5 by 11.58 percentage points with fewer parameters and comparable computational cost. Compared with YOLOv10n and YOLOv11n, TEA-LWNet improved mAP@0.5 by 7.08 and 5.28 percentage points, respectively. Although DEMNet also showed competitive performance, TEA-LWNet still achieved a 3.88 percentage-point improvement in mAP@0.5 with fewer parameters. These results indicate that the proposed lightweight multi-scale feature extraction and fusion design is effective for weakly contrasted tea disease regions and small pest-damage targets under comparable computational budgets.

Larger detectors and restoration-based pipelines were not included in the main comparison because their parameter scales, computational costs, and processing workflows differ substantially from those of lightweight detectors. Therefore, the revised comparison focuses on models with more comparable computational budgets.

**Figure 14:**
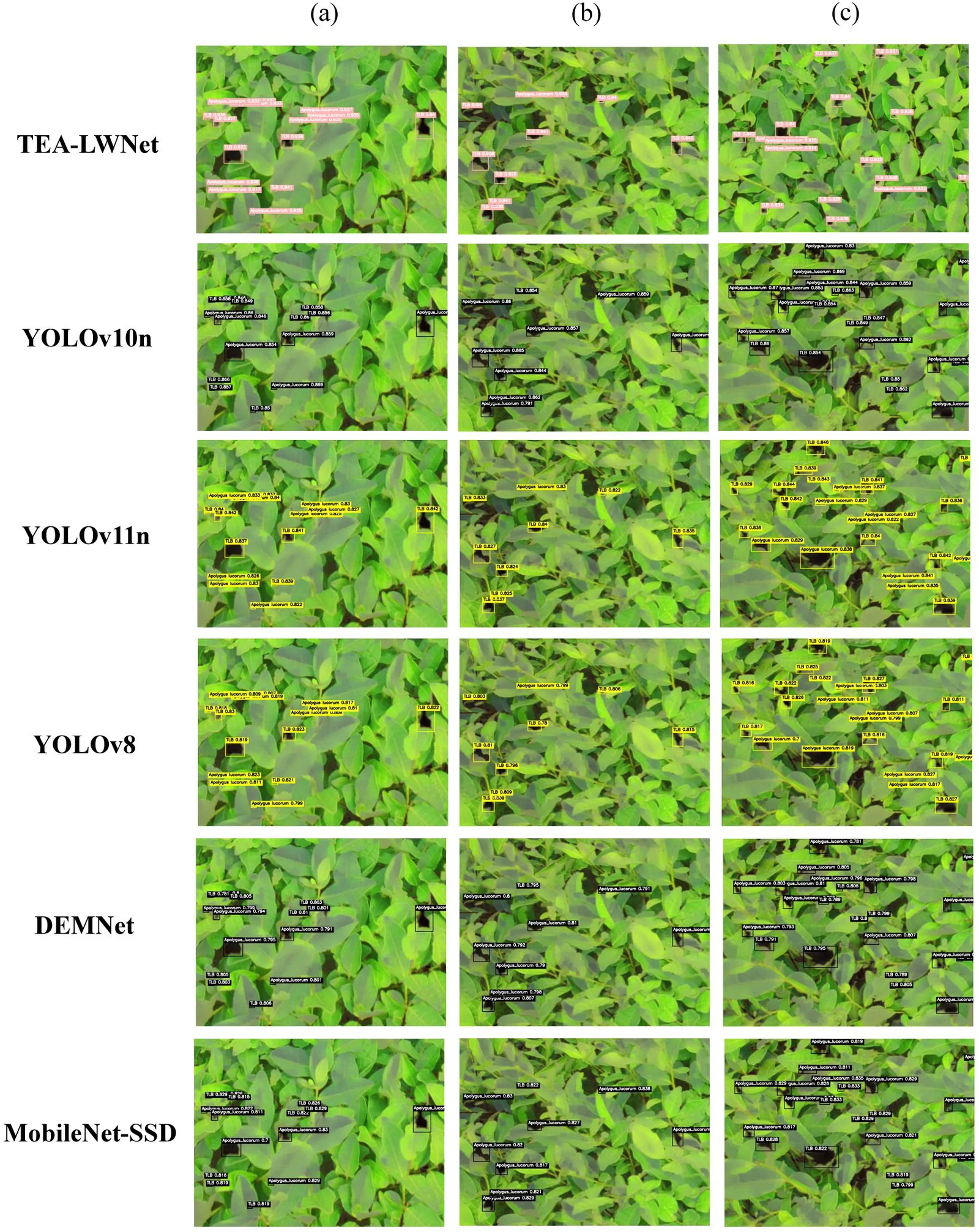
Qualitative visualization of comparative detection results on multi-angle field images. **(a)** Low-altitude UAV-acquired sample. **(b)** Sample captured under low-illumination conditions. **(c)** Sample with cluttered/complex background.

The visualization results are shown in **Figure 14**, which presents qualitative examples selected from multi-angle images and intuitively corroborates the quantitative analysis. In challenging scenarios characterized by severe occlusion from the leaf canopy, weak target texture, and low contrast, some lightweight baseline detectors exhibit higher rates of missed detections or false positives. In contrast, the proposed TEA-LWNet can more accurately locate and identify densely distributed small pest-damage and disease regions, and its detection boxes show higher overlap with the actual targets, validating its robustness in complex tea plantation environments. In challenging scenarios characterized by severe occlusion from the leaf canopy, weak target texture, and low contrast (as shown in the rows of the figure), some general-purpose detectors or restoration methods exhibit higher rates of missed detections or false positives. In contrast, the proposed TEA-LWNet is able to more accurately locate and identify densely distributed small target diseases, and its detection boxes have a higher overlap with the actual lesions, validating its robustness in complex tea plantation environments.

Combining the quantitative metrics in **Table 6** and the qualitative results in **Figure 14**, TEA-LWNet shows a favorable balance between detection accuracy and model complexity among the compared lightweight detectors. The relatively low parameter count and computational cost indicate its potential for resource-constrained scenarios, although further validation on real edge devices is still needed. Compared with the lightweight baseline models, TEA-LWNet performs better in detecting small targets, weak textures, and mildly blurred regions. The RGB-NIR fused representation further enhances target-background contrast, which helps improve detection under complex tea canopy conditions.

### 4.3 Comparison of RGB–NIR fusion strategies

To investigate the contribution of NIR information and justify the selection of the HSV-Brovey strategy, four input schemes were compared: RGB-only input, direct RGB-NIR channel concatenation followed by a 1 × 1 convolution, wavelet fusion, and HSV-Brovey fusion. For direct concatenation, the registered NIR image was concatenated with the three RGB channels to form a four-channel tensor, and a 1 × 1 convolution was used to project it into a representation compatible with the detector. Wavelet fusion combined the registered RGB and NIR information in the wavelet domain and reconstructed a three-channel fused image. HSV-Brovey fusion incorporated the registered NIR response into the value channel while retaining the hue and saturation information of the RGB image. All schemes used the same TEA-LWNet backbone, neck, detection head, dataset partition, training strategy, input resolution, and evaluation thresholds. Only the input-construction strategy differed. The direct-concatenation baseline additionally employed a lightweight 1 × 1 projection to adapt the four-channel RGB-NIR representation to the detector. The quantitative results are presented in **Table 7**.

**Table 7:** Comparison of different RGB-NIR fusion strategies under the same training and evaluation settings.

Fusion strategy	Fusion input channels	P/%	R/%	mAP@0.5/%	GFLOPs	Params/M
RGB only	3	84.6	82.3	89.40	7.63	1.64
Direct concatenation + 1×1 convolution	4	86.3	85.7	91.60	7.68	1.65
Wavelet fusion	3	87.5	88.1	92.90	7.63	1.64
HSV-Brovey fusion	3	88.6	89.4	93.48	7.63	1.64

As shown in **Table 7**, all three RGB-NIR fusion strategies outperformed the RGB-only baseline, indicating that NIR information provides complementary cues beyond visible color and texture. Direct channel concatenation achieved a Precision of 86.3%, a Recall of 85.7%, and an mAP@0.5 of 91.60%, improving mAP@0.5 by 2.20 percentage points over RGB-only input. However, the additional 1 × 1 projection increased the computational cost from 7.63 to 7.68 GFLOPs and the parameter count from 1.64M to 1.65M.

Wavelet fusion further increased mAP@0.5 to 92.90%, exceeding direct concatenation by 1.30 percentage points without changing the parameter count or detector GFLOPs. HSV-Brovey fusion achieved the best overall results, with a Precision of 88.6%, a Recall of 89.4%, and an mAP@0.5 of 93.48%. Compared with the RGB-only baseline, these values represent improvements of 4.0, 7.1, and 4.08 percentage points, respectively. HSV-Brovey fusion also exceeded wavelet fusion by 0.58 percentage points in mAP@0.5 while retaining the same detector complexity.

**Figure 15:**
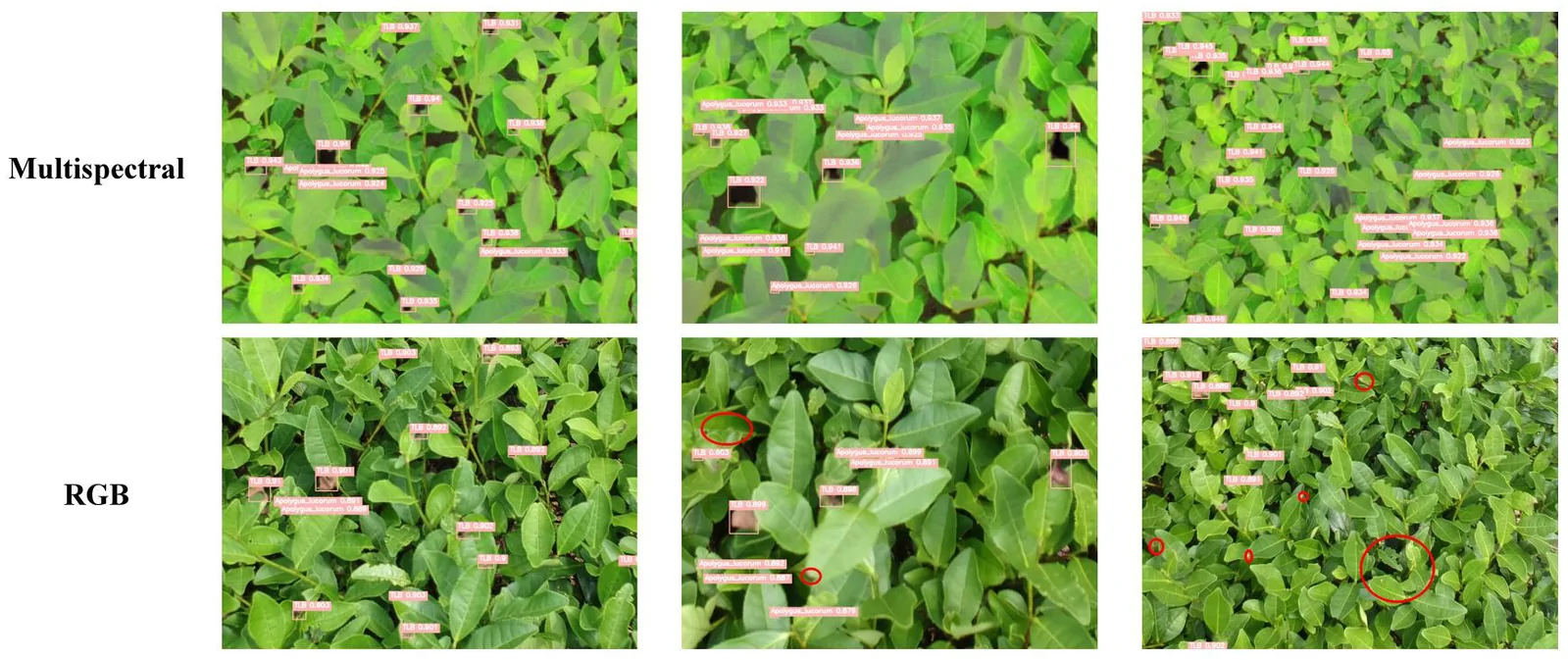
Visual comparison between RGB input and RGB–NIR input. The red circles indicate missed detections in the RGB-only result.

As shown in **Figure 15**, even under challenging conditions such as highly similar leaf textures, weak target-background contrast, and local mild blur, the model using HSV-Brovey fused input can locate targets more reliably than the RGB-only model. In contrast, the RGB-only model exhibits a higher failure probability in similar scenarios. For Apolygus lucorum, the fused input improves the visibility of small punctate feeding traces, local perforations, damaged leaf margins, and scattered necrotic spots. These symptoms are usually small in size and are easily confused with leaf veins, shadows, or background noise in RGB images. After introducing the NIR channel, the local contrast between damaged tissues and surrounding healthy leaf areas is enhanced, making small feeding traces and discontinuous edges more distinguishable.

In contrast, TLB generally appears as larger irregular or flaky lesions, often accompanied by gradual color transition, tissue necrosis, and blurred lesion boundaries. Compared with pest feeding traces, TLB regions usually show more continuous spatial distribution and stronger regional texture changes. The NIR channel provides complementary information related to tissue structure and water-status variation, which helps highlight lesion regions and suppress illumination-induced false responses. Therefore, RGB information mainly supports the recognition of color, shape, and texture differences, while NIR information enhances the separability of damaged tissues from healthy leaf backgrounds. The fused RGB–NIR representation enables the model to distinguish small punctate pest damage from larger continuous disease lesions more effectively.

It should be noted that pest-induced perforations are not equivalent to inter-leaf gaps or background voids. Apolygus lucorum damage usually appears as small, irregular, and discontinuous feeding traces located on the leaf surface or along damaged leaf margins, and the surrounding leaf tissue often shows local necrosis or structural disruption. In contrast, inter-leaf gaps are mainly formed by canopy overlap and leaf arrangement, usually showing larger open regions with clearer background exposure and without adjacent damaged tissue responses. In the fused images, damaged leaf areas retain leaf-surface reflectance, whereas inter-leaf gaps mainly expose background or shadow. RGB–NIR fusion therefore allows the model to jointly use visible morphology and measured NIR contrast, reducing confusion between true pest feeding traces and gap-like background patterns. This image-level interpretation should not be read as evidence of an *Apolygus lucorum*-specific reflectance signature at 860 nm.

Overall, HSV-Brovey fusion provided the most favorable performance among the evaluated strategies. Unlike direct channel concatenation, it did not require an additional learnable input-adaptation layer or increase the parameter count and GFLOPs of the detector. Its performance advantage over wavelet fusion was moderate, with an improvement of 0.58 percentage points in mAP@0.5. Therefore, HSV-Brovey fusion was selected as a practical deterministic preprocessing strategy based on its favorable detection performance, input compatibility, and computational simplicity, rather than being treated as a newly proposed learnable fusion module.

### 4.4 Ablation experiments

**Figure 16:**
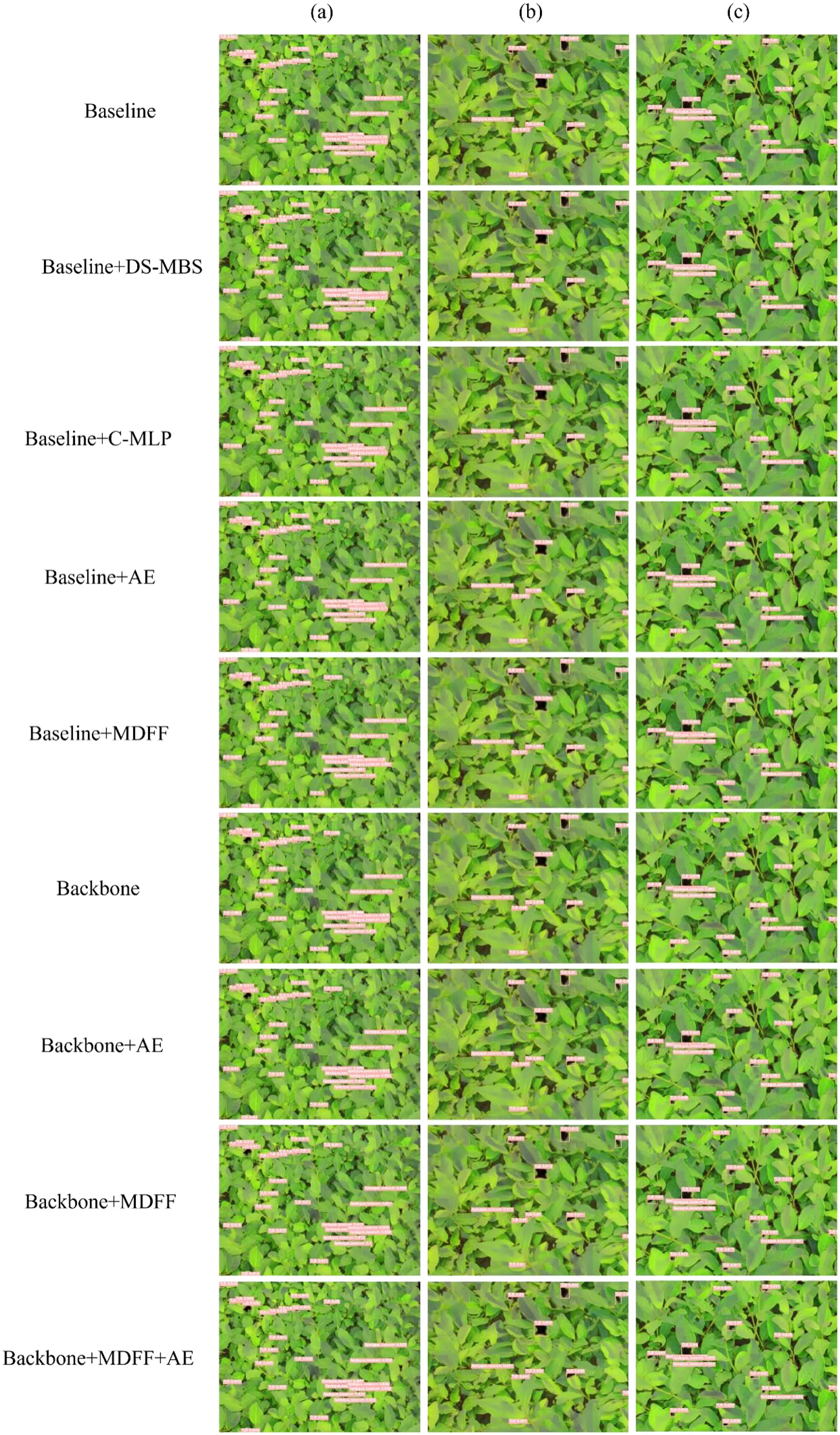
Qualitative visualization of ablation study results on multi-angle field images. **(a)** Low-altitude UAV-acquired sample. **(b)** Sample captured under low-illumination conditions. **(c)** Sample with cluttered/complex background.

In this study, the baseline model replaces the proposed DS-MBS and C-MLP modules in the backbone with standard convolutional layers, while retaining the basic feature transfer path. Unless otherwise specified in **Table 8**, the other components are kept unchanged to ensure controlled comparisons. All experiments were conducted under a unified training and evaluation pipeline to ensure fair comparison. The performance comparison of different module combinations is shown in **Table 8** and **Figure 16**.

**Table 8:** Ablation experiments data comparison table.

Baseline	DS-MBS	C-MLP	AE	MDFF	P/%	R/%	mAP@0.5/%	F1/%
✓					78.3	76.8	80.1	77.54
✓	✓				79.5	77.1	82.9	78.28
✓		✓			78.9	75.2	81.5	77.01
✓			✓		82.7	80.1	85.6	81.38
✓				✓	87.6	85.5	90.1	86.54
✓	✓	✓			84.2	82.8	87.8	83.49
✓	✓	✓	✓		87.9	85.2	90.7	86.53
✓	✓	✓		✓	89.3	87.9	91.2	88.59
✓	✓	✓	✓	✓	88.6	89.4	93.48	89

The baseline model is used to characterize the detection capability without the proposed structural reconstruction and channel modeling modules. Its performance metrics are P = 78.3%, R = 76.8%, mAP@0.5 = 80.1%, and F1 = 77.54%.

The results in **Table 8** show the contribution of each module to detection performance. When individual modules were introduced, different levels of improvement were observed. The DS-MBS module increased mAP@0.5 by 2.8 percentage points, reaching 82.9%, by improving multi-scale representation and reducing redundant computation. The C-MLP module increased mAP@0.5 by 1.4 percentage points, reaching 81.5%, indicating that channel interaction contributes to feature refinement, although recall was slightly reduced. Among the individual modules, MDFF achieved the largest improvement in mAP@0.5, while AE also provided a clear performance gain. Specifically, AE strengthened the model’s focus on weakly contrasted and fine-grained regions, increasing mAP@0.5 by 5.5 percentage points to 85.6% and improving F1 by 3.84 percentage points.

When DS-MBS and C-MLP were jointly introduced, mAP@0.5 reached 87.8%, showing that multi-scale feature extraction and channel refinement are complementary. After AE was further added, mAP@0.5 increased to 90.7%, confirming the benefit of spatial–semantic enhancement based on improved backbone features. When MDFF was introduced on the basis of DS-MBS and C-MLP, mAP@0.5 reached 91.2% and F1 reached 88.59%, suggesting that bidirectional feature fusion further improves recall and overall detection stability.

When DS-MBS, C-MLP, AE, and MDFF were all enabled, the model achieved its final performance, with P = 88.6%, R = 89.4%, mAP@0.5 = 93.48%, and F1 = 89.00%. Compared with the baseline, the complete model improved P, R, mAP@0.5, and F1 by 10.3, 12.6, 13.38, and 11.46 percentage points, respectively. These results indicate that DS-MBS and C-MLP provide efficient feature enhancement in the backbone, while AE and MDFF improve spatial–semantic consistency and multi-scale feature fusion in the neck. The modules work together to improve both detection accuracy and recall.

### 4.5 Cross-regional dataset validation experiments

To evaluate the generalization capability and practical applicability of the proposed model across diverse tea plantation scenarios, this study used datasets collected from five geographically distinct tea-producing regions. The training data were acquired from Fulier Tea Plantation in Wuyishan, Nanping, Fujian Province; Lianhuadi Eco-Tea Garden in Huoshan County, Lu’an, Anhui Province; and a tea cultivation base in Lanshan District, Rizhao, Shandong Province. The cross-regional validation data were acquired from Maoshan Tea Garden in Jurong City, Zhenjiang, Jiangsu Province, and Yuewan Tea Garden in Qiukou Town, Wuyuan County, Jiangxi Province. These two validation sites were not included in model training and were used to evaluate the generalization ability of TEA-LWNet under unseen regional conditions.

The two cross-regional validation sites exhibit differences in terrain, canopy structure, illumination conditions, and background complexity, thereby providing representative scenarios for examining model robustness under varying natural environments and cultivation conditions. To increase sample diversity, multi-angle imaging strategies were adopted at the validation sites, including nadir views, oblique perspectives, and low-altitude close-range shots. This acquisition design covered variations in illumination direction, leaf pose, and occlusion, thus better approximating real-world field monitoring conditions. Although regional differences may lead to variations in disease appearance, samples with similar visual symptoms were annotated under the TLB category according to the annotation protocol used in this study.

**Figure 17:**
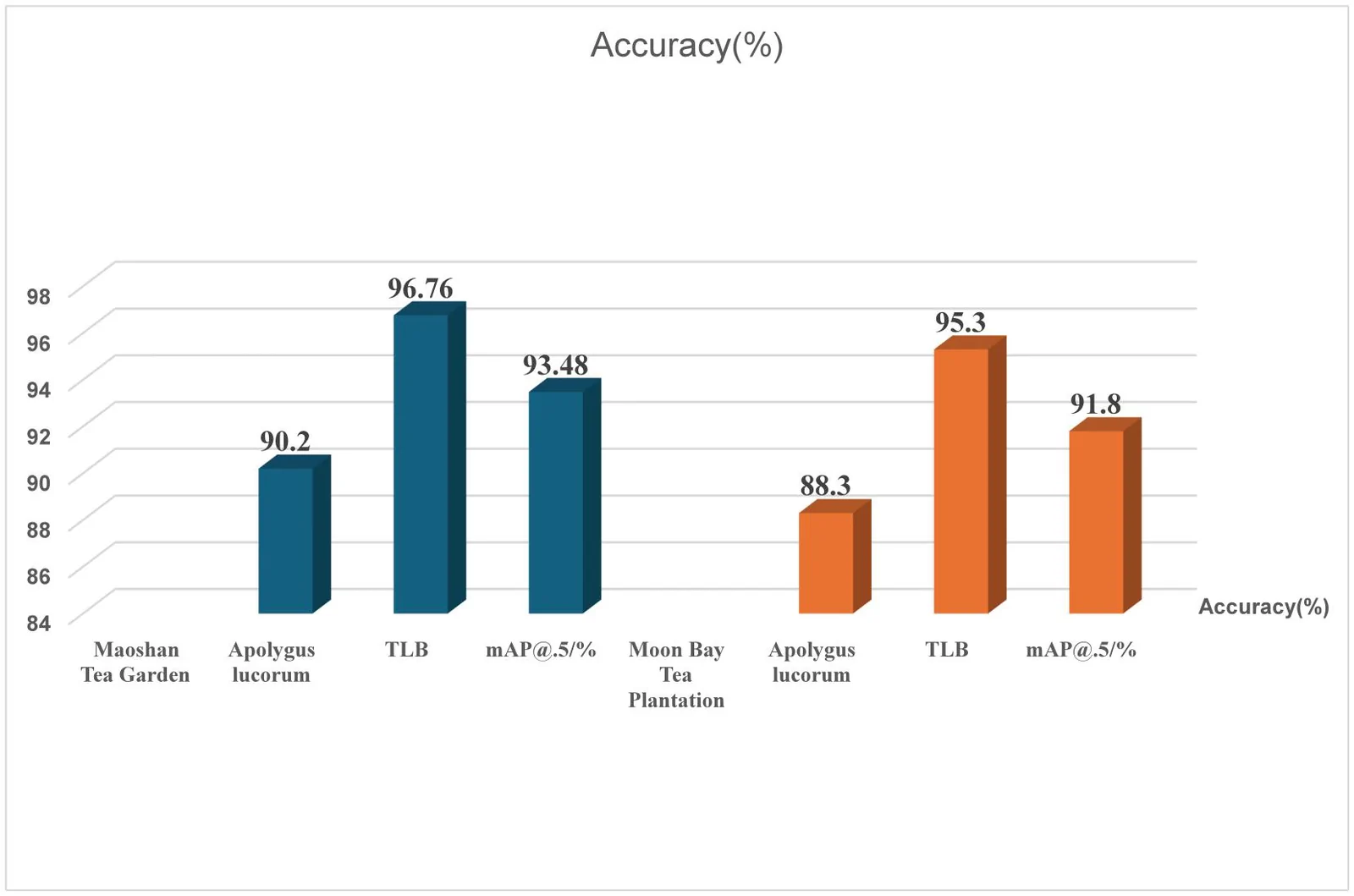
Bar chart of comparative results for cross-regional dataset validation experiments.

The cross-regional validation results are presented in **Figure 17**. On the Maoshan validation dataset, the proposed model achieved AP@0.5 values of 90.2% and 96.76% for Apolygus lucorum and TLB, respectively, with an overall mAP@0.5 of 93.48%. On the Yuewan validation dataset, the model obtained AP@0.5 values of 88.3% and 95.3% for the two categories, with an overall mAP@0.5 of 91.8%. The comparable results across the two validation sites indicate that the model maintains stable recognition capability in cross-regional scenarios and demonstrates good adaptability to variations in viewing angles and complex backgrounds, thereby suggesting promising generalization potential across different tea-producing regions.

## 5 Discussion and future work

The TEA-LWNet framework achieves accurate small-target detection in tea plantations with a lightweight RGB–NIR fusion design, reaching an mAP@0.5 of 93.48% in the experiments. The framework uses Multi-FENet with DS-MBS to preserve fine-grained details, and uses AE and MDFF to improve spatial–semantic consistency and multi-scale feature fusion, with GF controlling feature injection in MDFF. SAB-Loss further supports boundary refinement and classification stability. Ablation studies confirm the functional complementarity of these modules in noise suppression and feature alignment. An important limitation of this study is that only one broad NIR band centered at 860 ± 26 nm was used, without collecting leaf-level hyperspectral reflectance curves or concurrent measurements of pigment content, water status, and tissue structure. Therefore, the performance improvement obtained through RGB–NIR fusion cannot be assigned to a target-specific response at 860 nm. Future work will acquire calibrated spectra from healthy, TLB-affected, and *Apolygus lucorum*-damaged leaves, quantify reflectance differences and effect sizes across the 834–886 nm passband, and evaluate whether 860 nm is optimal relative to adjacent red-edge and NIR wavelengths.

Future work will proceed in three directions. Regarding data, hyperspectral UAV data will be introduced to construct spectral-index representations, such as NDVI and SAVI, and semi-supervised learning will be explored to improve generalization. Regarding the model, spectral attention, channel re-weighting, and dynamic convolution will be further investigated. In addition, cross-modal fusion between RGB and real multispectral data will be studied so that detection results can be displayed directly on standard RGB streams, thereby improving visual interpretability and human–machine interaction. Regarding deployment, structural re-parameterization, INT8 quantization, and TensorRT optimization will be implemented on platforms such as Jetson for real-time inference. These future efforts are expected to further improve the balance between accuracy and deployability.

## Data Availability

The raw data supporting the conclusions of this article will be made available by the authors, without undue reservation. The official implementation of TEA-LWNet is available at https://github.com/zhenyunw00-lab/TEA-LWNet.git.

## References

[B1] Bao W. Fan T. Hu G. Liang D. Li H. (2022). Detection and identification of tea leaf diseases based on AX-RetinaNet. Sci. Rep. 12, 2183. doi: 10.1038/s41598-022-06181-z 35140287 PMC8828751

[B2] Bao W. Zhu Z. Hu G. Zhou X. Zhang D. Yang X. (2023). UAV remote sensing detection of tea leaf blight based on DDMA-YOLO. Comput. Electron. Agric. 205, 107637. doi: 10.1016/j.compag.2023.107637 38826717

[B3] Bhuyan P. Singh P. K. Das S. K. (2024). Res4net-CBAM: a deep cnn with convolution block attention module for tea leaf disease diagnosis. Multimed. Tools Appl. 83, 48925–48947. doi: 10.1007/s11042-023-17472-6 30311153

[B4] Chen Z. He Z. Lu Z.-M. (2024). DEA-Net: Single image dehazing based on detail-enhanced convolution and content-guided attention. IEEE Trans. Img. Process. 33, 1002–1015. doi: 10.1109/TIP.2024.3354108 38252568

[B5] Dalgaty T. Moro F. Demirag Y. De Pra A. Indiveri G. Vianello E. . (2024). Mosaic: in-memory computing and routing for small-world spike-based neuromorphic systems. Nat. Commun. 15, 142. doi: 10.1038/s41467-023-44365-x 38167293 PMC10761708

[B6] Duan Z. Li H. Li C. Zhang J. Zhang D. Fan X. . (2024). A CNN model for early detection of pepper Phytophthora blight using multispectral imaging, integrating spectral and textural information. Plant Methods 20, 115. doi: 10.1186/s13007-024-01239-7 39075512 PMC11288097

[B7] Hogräfer M. Burkhardt J. Schulz H.-J. (2022). “ A pipeline for tailored sampling for progressive visual analytics”, in: Eurovis Workshop on Visual Analytics, Eurova 2022 (Aire-La-Ville: Eurographics Assoc), 49–53. doi: 10.2312/eurova.20221079

[B8] Hu G. Ye R. Wan M. Bao W. Zhang Y. Zeng W. (2024). Detection of tea leaf blight in low-resolution UAV remote sensing images. IEEE Trans. Geosci. Remote Sens. 62, 5601218. doi: 10.1109/TGRS.2023.3339765 25079929

[B9] Hu Y. Tan W. Meng F. Liang Y. (2023). “ A decoupled spatial-channel inverted bottleneck for image compression”, in: 2023 IEEE International Conference on Image Processing, ICIP (New York: IEEE), 1740–1744. doi: 10.1109/ICIP49359.2023.10222366

[B10] Ismagilov T. Ferrarini B. Milford M. Nguyen T. V. T. Ramchurn S. D. Ehsan S. (2025). On motion blur and deblurring in visual place recognition. IEEE Robot. Autom. Lett. 10, 4746–4753. doi: 10.1109/LRA.2025.3554103

[B11] Ke H. Li H. Wang B. Tang Q. Lee Y.-H. Yang C.-F. (2024). Integrations of LabelImg, You Only Look Once (YOLO), and Open Source Computer Vision Library (OpenCV) for chicken open mouth detection. Sens. Mater. 36, 4903–4913. doi: 10.18494/SAM5108

[B12] Lei M. Li S. Wu Y. Hu H. Zhou Y. Zheng X. . (2025). YOLOv13: real-time object detection with hypergraph-enhanced adaptive visual perception. arXiv [Preprint], arXiv:2506.17733. doi: 10.48550/arXiv.2506.17733

[B13] Li X. Wang W. Wu L. Chen S. Hu X. Li J. . (2020). Generalized focal loss: learning qualified and distributed bounding boxes for dense object detection. Adv. Neural Inf. Process. Syst. 33, 21002–21012.

[B14] Lu J. Luo H. Yu C. Liang X. Huang J. Wu H. . (2024). Tea bud DG: a lightweight tea bud detection model based on dynamic detection head and adaptive loss function. Comput. Electron. Agric. 227, 109522. doi: 10.1016/j.compag.2024.109522

[B15] Mao Y. Li H. Xu Y. Wang S. Yin X. Fan K. . (2024). Early detection of gray blight in tea leaves and rapid screening of resistance varieties by hyperspectral imaging technology. J. Sci. Food Agric. 104, 9336–9348. doi: 10.1002/jsfa.13756 39030928

[B16] Medina J. Becerra Gonzalez L. Upegui E. (2019). “ Spectral and spatial assessment of the Ikonos images fusion, using the Principal Components, Brovey Transform and Multiplicative”, in: 2019 14th Iberian Conference on Information Systems and Technologies (CISTI) (New York: IEEE).

[B17] Mohammadi N. Esteki M. Simal-Gandara J. (2024). Machine learning for authentication of black tea from narrow-geographic origins: Combination of PCA and PLS with LDA and SVM classifiers. LWT Food Sci. Technol. 203, 116401. doi: 10.1016/j.lwt.2024.116401 38826717

[B18] Mora J. J. Selvaraj M. G. Alvarez C. I. Safari N. Blomme G. (2024). From pixels to plant health: accurate detection of banana Xanthomonas wilt in complex African landscapes using high-resolution UAV images and deep learning. Discov. Appl. Sci. 6, 377. doi: 10.1007/s42452-024-06073-z 30311153

[B19] Pan Z. Gu J. Wang W. Fang X. Xia Z. Wang Q. . (2024). Picking point identification and localization method based on Swin-Transformer for high-quality tea. J. King Saud Univ. Comput. Inf. Sci. 36, 102262. doi: 10.1016/j.jksuci.2024.102262

[B20] Shang C. Zhou S. Zhang H. Ni X. Yang Y. Wang Y. (2024). “ Incremental residual concept bottleneck models”, in: 2024 IEEE/CVF Conference on Computer Vision and Pattern Recognition (CVPR) (Los Alamitos: IEEE Computer Soc), 11030–11040. doi: 10.1109/CVPR52733.2024.01049

[B21] Tolstikhin I. O. Houlsby N. Kolesnikov A. Beyer L. Zhai X. Unterthiner T. . (2021). MLP-Mixer: an all-MLP architecture for vision. Adv. Neural Inf. Process. Syst. 34, 24261–24272.

[B22] Wei K. Dai J. Hong D. Ye Y. (2024). MGFNet: An MLP-dominated gated fusion network for semantic segmentation of high-resolution multi-modal remote sensing images. Int. J. Appl. Earth Obs. Geoinf. 135, 104241. doi: 10.1016/j.jag.2024.104241 38826717

[B23] Wu W. Tang T. Duan Y. Qiu W. Duan L. Lv J. . (2025). Study on the detection model of tea red scab severity class using hyperspectral imaging technology. Agri. 15, 2372. doi: 10.3390/agriculture15222372

[B24] Xia Y. Yuan W. Zhang S. Wang Q. Liu X. Wang H. . (2024). Classification and identification of tea diseases based on improved YOLOv7 model of MobileNeXt. Sci. Rep. 14, 11799. doi: 10.1038/s41598-024-62451-y 38782981 PMC11116536

[B25] Xie X. Yue C. He W. Peng H. Wang Z. Peng Y. . (2026). Physical and chemical properties of different tea germplasms damaged by Apolygus lucorum. Acta Agric. Jiangxi 38, 76–83. doi: 10.19386/j.cnki.jxnyxb.2026.04.010

[B26] Xiong B. Li D. Zhang Q. Desneux N. Luo C. Hu Z. (2024). Image detection model construction of Apolygus lucorum and Empoasca spp. based on improved YOLOv5. Pest Manage. Sci. 80, 2577–2586. doi: 10.1002/ps.7964 38243837

[B27] Xu Y. Mao Y. Li H. Shen J. Xu X. Wang S. . (2025). A deep learning model based on RGB and hyperspectral images for efficiently detecting tea green leafhopper damage symptoms. Smart Agric. Technol. 10, 100817. doi: 10.1016/j.atech.2025.100817

[B28] Yang H.-M. Zhang X.-Y. Yin F. Liu C.-L. (2018). Robust classification with convolutional prototype learning. Available online at: https://openaccess.thecvf.com/content_cvpr_2018/html/Yang_Robust_Classification_With_CVPR_2018_paper.html (Accessed November 10, 2025).

[B29] Ye R. Gao Q. Qian Y. Sun J. Li T. (2024a). Improved YOLOv8 and SAHI model for the collaborative detection of small targets at the micro scale: A case study of pest detection in tea. Agronomy-Basel 14, 1034. doi: 10.3390/agronomy14051034 30654563

[B30] Ye R. Shao G. He Y. Gao Q. Li T. (2024b). YOLOv8-RMDA: Lightweight YOLOv8 network for early detection of small target diseases in tea. Sensors 24, 2896. doi: 10.3390/s24092896 38733002 PMC11086262

[B31] Yin N. Bao W. Yang R. Wang N. Liu W. (2024). LWSDNet: A lightweight wheat scab detection network based on UAV remote sensing images. Remote Sens. 16, 2820. doi: 10.3390/rs16152820 30654563

[B32] Zhang H. Zhang S. (2024). Focaler-IoU: More focused intersection over union loss. arXiv:2401.10525. doi: 10.48550/arXiv.2401.10525

[B33] Zheng Z. Wang P. Liu W. Li J. Ye R. Ren D. . (2020). Distance-IoU loss: faster and better learning for bounding box regression. Proc. AAAI Conf. Artif. Intell. 34, 12993–13000. doi: 10.1609/aaai.v34i07.6999

[B34] Zhou Q. Shi H. Xiang W. Kang B. Latecki L. J. (2025). DPNet: Dual-path network for real-time object detection with lightweight attention. IEEE Trans. Neural Netw. Learn. Syst. 36, 4504–4518. doi: 10.1109/TNNLS.2024.3376563 38536700

